# Microbiome-mediated neutrophil recruitment via CXCR2 and protection from amebic colitis

**DOI:** 10.1371/journal.ppat.1006513

**Published:** 2017-08-17

**Authors:** Koji Watanabe, Carol A. Gilchrist, Md Jashim Uddin, Stacey L. Burgess, Mayuresh M. Abhyankar, Shannon N. Moonah, Zannatun Noor, Jeffrey R. Donowitz, Brittany N. Schneider, Tuhinur Arju, Emtiaz Ahmed, Mamun Kabir, Masud Alam, Rashidul Haque, Patcharin Pramoonjago, Borna Mehrad, William A. Petri

**Affiliations:** 1 Division of Infectious Diseases and International Health, Department of Medicine, University of Virginia, Charlottesville, Virginia, United States of America; 2 AIDS Clinical Center, National Center for Global Health and Medicine, Shinjuku, Tokyo, Japan; 3 International Centre for Diarrhoeal Disease Research, Bangladesh (icddr, b), Dhaka, Bangladesh; 4 Division of Pediatrics, Children’s Hospital of Richmond at Virginia Commonwealth University, Richmond, Virginia, United States of America; 5 Biorepository and Tissue Research Facility, University of Virginia School of Medicine, Charlottesville, Virginia, United States of America; 6 Division of Pulmonary and Critical Care Medicine, Department of Medicine, University of Virginia, Charlottesville, Virginia, United States of America; Stanford University School of Medicine, UNITED STATES

## Abstract

The disease severity of *Entamoeba histolytica* infection ranges from asymptomatic to life-threatening. Recent human and animal data implicate the gut microbiome as a modifier of *E*. *histolytica* virulence. Here we have explored the association of the microbiome with susceptibility to amebiasis in infants and in the mouse model of amebic colitis. Dysbiosis occurred symptomatic *E*. *histolytica* infection in children, as evidenced by a lower Shannon diversity index of the gut microbiota. To test if dysbiosis was a cause of susceptibility, wild type C57BL/6 mice (which are innately resistant to *E*. *histiolytica* infection) were treated with antibiotics prior to cecal challenge with *E*. *histolytica*. Compared with untreated mice, antibiotic pre-treated mice had more severe colitis and delayed clearance of *E*. *histolytica*. Gut IL-25 and mucus protein Muc2, both shown to provide innate immunity in the mouse model of amebic colitis, were lower in antibiotic pre-treated mice. Moreover, dysbiotic mice had fewer cecal neutrophils and myeloperoxidase activity. Paradoxically, the neutrophil chemoattractant chemokines CXCL1 and CXCL2, as well as IL-1β, were higher in the colon of mice with antibiotic-induced dysbiosis. Neutrophils from antibiotic pre-treated mice had diminished surface expression of the chemokine receptor CXCR2, potentially explaining their inability to migrate to the site of infection. Blockade of CXCR2 increased susceptibility of control non-antibiotic treated mice to amebiasis. In conclusion, dysbiosis increased the severity of amebic colitis due to decreased neutrophil recruitment to the gut, which was due in part to decreased surface expression on neutrophils of CXCR2.

## Author summary

Amebiasis, caused by intestinal infection of *Entamoeba histolytica*, is one of the leading causes of parasite infection-related mortality and morbidity around the world. However, pathogenesis, such as determinant factors of infection outcome, is still unclear although recent data indicate that the gut microbiome plays an important role. In the present study, we firstly found that dysbiosis, which was represented by a lower Shannon diversity index of the gut microbiota, occurred symptomatic *E*. *histolytica* infection in children living in endemic area. In mouse model, we demonstrated that dysbiosis induced by antibiotic pre-treatment increased the severity of amebic colitis due to decreased neutrophil activity as well as decreased IL-25 associated mucosal defense in the gut. Moreover, we demonstrated surface expression on neutrophils of CXCR2 was diminished in mice with dysbiosis, which resulted in decreased neutrophil recruitment to the gut. This study is of fundamental importance in amebiasis research for the discovery of a mechanism of microbiome-mediated resistance to amebiasis via neutrophil trafficking to the gut. The work is importantly of broad interest in infectious diseases and immunology for the discovery that neutrophil mediated protection can be disturbed by dysbiosis.

## Introduction

Amebiasis, caused by intestinal infection of *Entamoeba histolytica*, is one of the leading causes of parasite infection-related mortality and morbidity around the world [1]. Although disease severity ranges from self-limited mild abdominal symptoms to life-threatening systemic disease, determinant factors of infection outcome are still undefined [2]. Even in the same patient, invasive symptomatic disease can develop after long-term asymptomatic colonization [3–5], and conversely patients with amebic liver abscess after medical treatment can be asymptomatic cyst passers [6]. These results suggest that not only the genetics of host and pathogen are important for determining clinical symptoms of infected individuals, but also the gut environment surrounding *E*. *histolytica*. In fact, recent human and animal data indicate that the gut microbiome plays an important role in the pathogenesis of *E*. *histolytica* infection. Our group reported that the presence of *Prevotella copri* in gut flora is associated with susceptibility of children to *E*. *histolytica* induced diarrheal disease [7]. Also, in an animal model, we demonstrated gut colonization with segmented filamentous bacterium exerts a protective effect via enhancing the induction of IL-23 in bone marrow-derived dendritic cells [8, 9]. It is of interest to us to better understand the impact of the gut microbiome on the severity of amebic colitis, potentially by its modulation of intestinal mucosal immunity.

Neutrophils are important in protecting the host from *E*. *histolytica* tissue invasion into intestine and liver [10–16]. Neutrophils kill *E*. *histolytica* in vitro in the presence of TNF-α and IFN-γ mainly via oxygen free radicals [17]. Antibody-depletion of neutrophils in vivo promoted tissue invasion by *E*. *histolytica* [14, 16], and neutrophil chemotaxis toward leptin plays an important role in protecting host from intestinal tissue invasion [16].

Dysbiosis is known to affect neutrophil function. For example, in an animal model it has been shown that the severity of sickle cell disease is relieved under antibiotic induced dysbiosis, due to a decrease in the number of activated aged neutrophils [18]. However the effect of dysbiosis on neutrophil mediated protection against infectious diseases has not been investigated.

Here we demonstrate the impact of dysbiosis on disease severity of *E*. *histolytica* infection in humans and in a mouse model of amebic colitis. We go on to demonstrate that one mechanism by which dysbiosis increases susceptibility is by blockading neutrophil recruitment to the gut via down-regulation of CXCR2.

## Results

### Decreased gut microbiota diversity in children who developed amebic colitis

**Fig 1:**
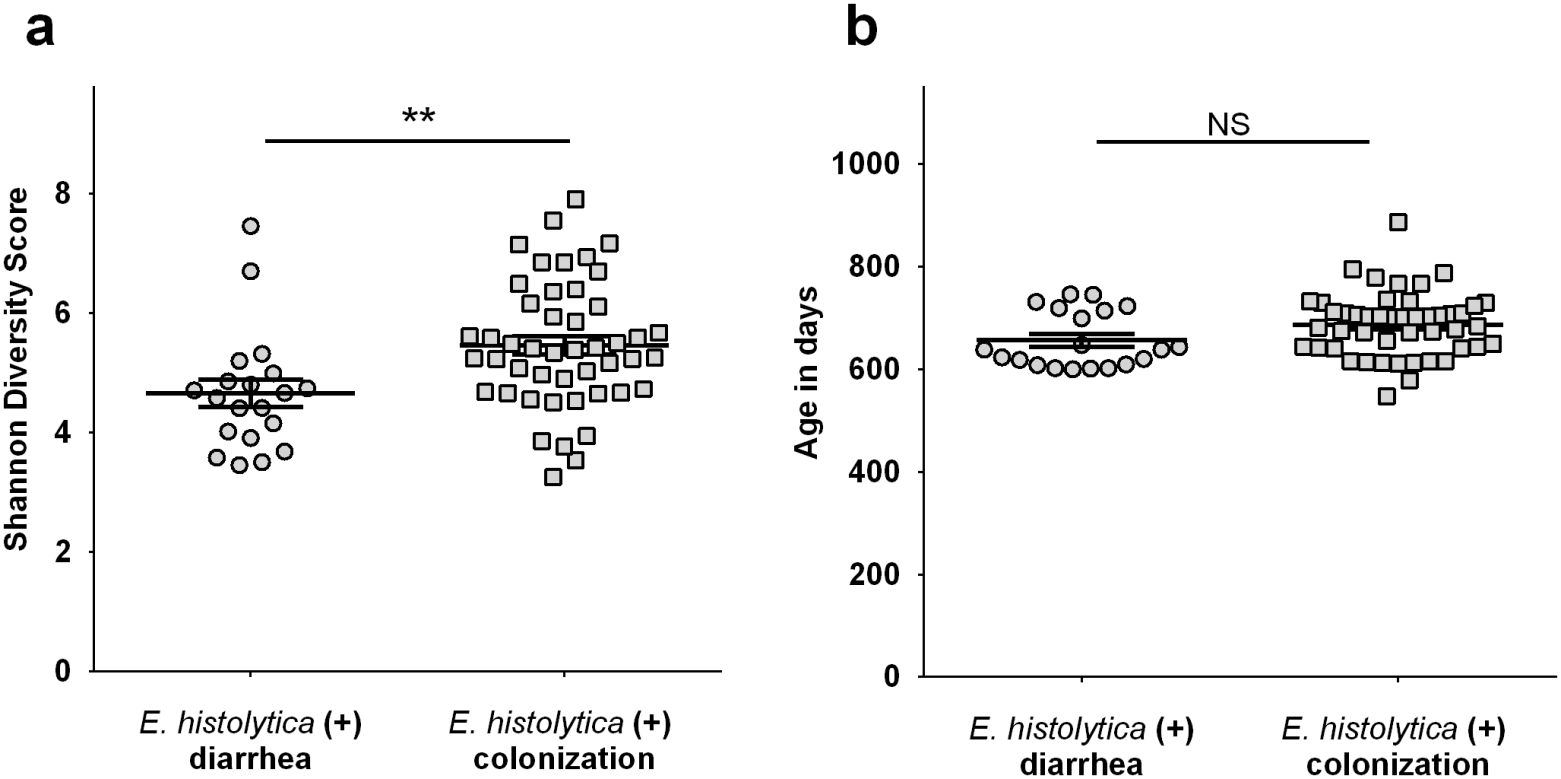
Diversity of microbiome was decreased during amebic colitis. Diarrhea samples were from children in PROVIDE study (n = 20) and colonization samples were from children in NIH birth cohort (n = 47), both of which were followed longitudinally in an urban slum in Dhaka, Bangladesh. **(a)** The Shannon diversity index was examined using stool samples between 2 groups. **(b)** Age in days when stool samples were collected was also compared. *P<0.*05*, by Welch’s unequal variance t-test. Error bars represent standard error of the mean (s.e.m).

In order to assess the impact of dysbiosis on outcome of intestinal infection of *E*. *histolytica*, we collected stool samples from children cohorts followed from birth in an urban slum, Mirpur in Dhaka Bangladesh [19, 20]. First, we compared gut microbiome diversity in stools collected from children with symptomatic amebic colitis with those from children who showed asymptomatic *E*. *histolytica* colonization. The Shannon diversity index during *E*. *histolytica* infection was lower in symptomatic cases than with colonization (Fig 1a). We confirmed that ages of children when stool samples were collected were not different between 2 groups (Fig 1b). Next, we examined microbiome diversity of stools prior to *E*. *histolytica* infection (no *E*. *histolytica* confirmed by PCR) in symptomatic amebic colitis cases, and compared them with uninfected control children (although we could not use stools prior to *E*. *histolytica* infection in asymptomatic colonization cases due to the lack of available samples). The Shannon diversity index prior to amebic colitis was significantly lower than that in children who did not develop *E*. *histolytica* infection (S1 Fig). Although other factors, such as host/pathogen genetic factors are likely also to be important determinants, our results suggest that decreased microbiota diversity in the gut is one of the determinants for disease severity of *E*. *histolytica* infection.

### Severity of amebic colitis in antibiotic pre-treated mice

**Fig 2:**
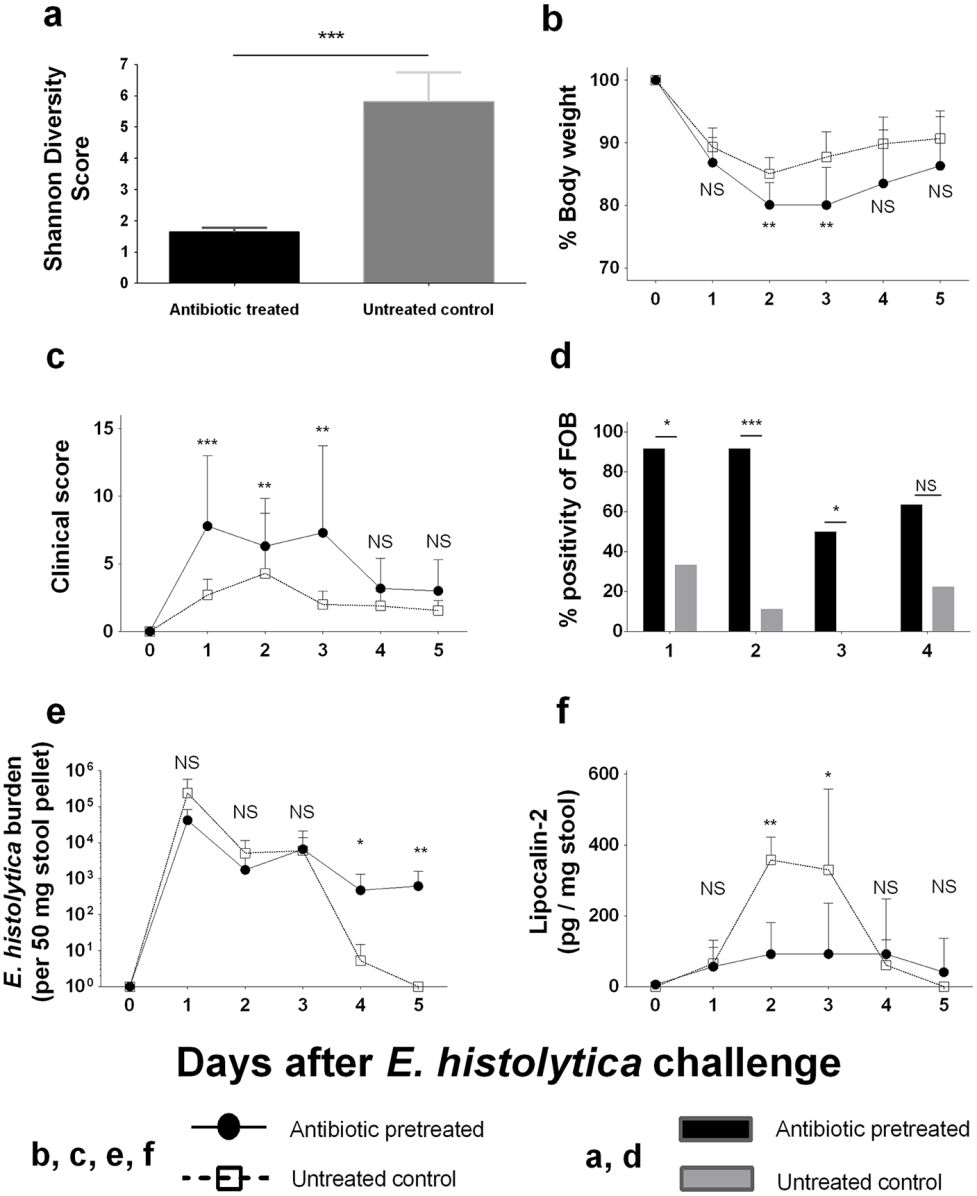
Antibiotic pre-treatment rendered C57BL/6 mice susceptible to *E*. *histolytica* colitis. **(a)** The Shannon diversity index of stool samples collected from antibiotic treated mice (n = 10) was compared with those from untreated control (n = 10) (C57BL/6 mice which were not challenged with *E*. *histolytica*). Then, they were infected with 2 x 10^6^ *E*. *histolytica* trophozoites intracecally. **(b, c)** Body weight changes and clinical illness score were measured for assessing systemic disease severity. **(d)** Fecal occult blood was examined for assessing intestinal damage. **(e)** Parasite burden in stool was assessed by qPCR. **(f)** Lipocalin-2 (Neutrophil Gelatinase-Associated Lipocalin) in stool was measured for assessing neutrophil mediated gut inflammation. *P<0.*05*, **P<0.*01*, ***P<0.*001* by Welch’s unequal variance t-test (a, b, e& f), Mann-Whitney U-test (c) or chi-squared test (d). NS, not significant. Error bars represent s.e.m.

We used the murine model of amebic colitis to assess whether prior dysbiosis with decreased microbiome diversity has an impact on disease severity of amebic colitis. Wild type C57BL/6 mice were treated with an antibiotic cocktail consisting of ampicillin, neomycin, metronidazole and vancomycin for 2 weeks prior to *E*. *histolytica* challenge (antibiotic pre-treated mice). We confirmed the microbiome diversity was decreased by antibiotic pre-treatment before *E*. *histolytica* challenge (Fig 2a). Antibiotic pre-treated mice showed more severe weight loss and higher clinical scores than untreated control mice at early time points (until day 3) after *E*. *histolytica* challenge (Fig 2b & 2c). Although obviously bloody stools were not documented in any mice, fecal occult blood (FOB) was significantly higher in antibiotic pre-treated mice (Fig 2d). Also, we found that *E*. *histolytica* DNA was still detected at later time points (after 4 days on challenge in stool [Fig 2e] and at day 9 in cecal contents [S3a Fig]) in antibiotic pre-treated mice, whereas *E*. *histolytica* was rapidly cleared in untreated control mice (Fig 2e). Interestingly, despite delayed clearance of *E*. *histolytica*, stool lipocalin-2, which is a neutrophil derived protein reflecting neutrophil associated gut inflammation [21], was lower in antibiotic pre-treated mice than those in untreated control mice at early time points of infection (Fig 2f). These results indicated that antibiotic-induced dysbiosis increased susceptibility to amebic colitis in the mouse model, consistent with what had been observed in infants.

### IL-25 and mucosal barrier in antibiotic pre-treated mice

**Fig 3:**
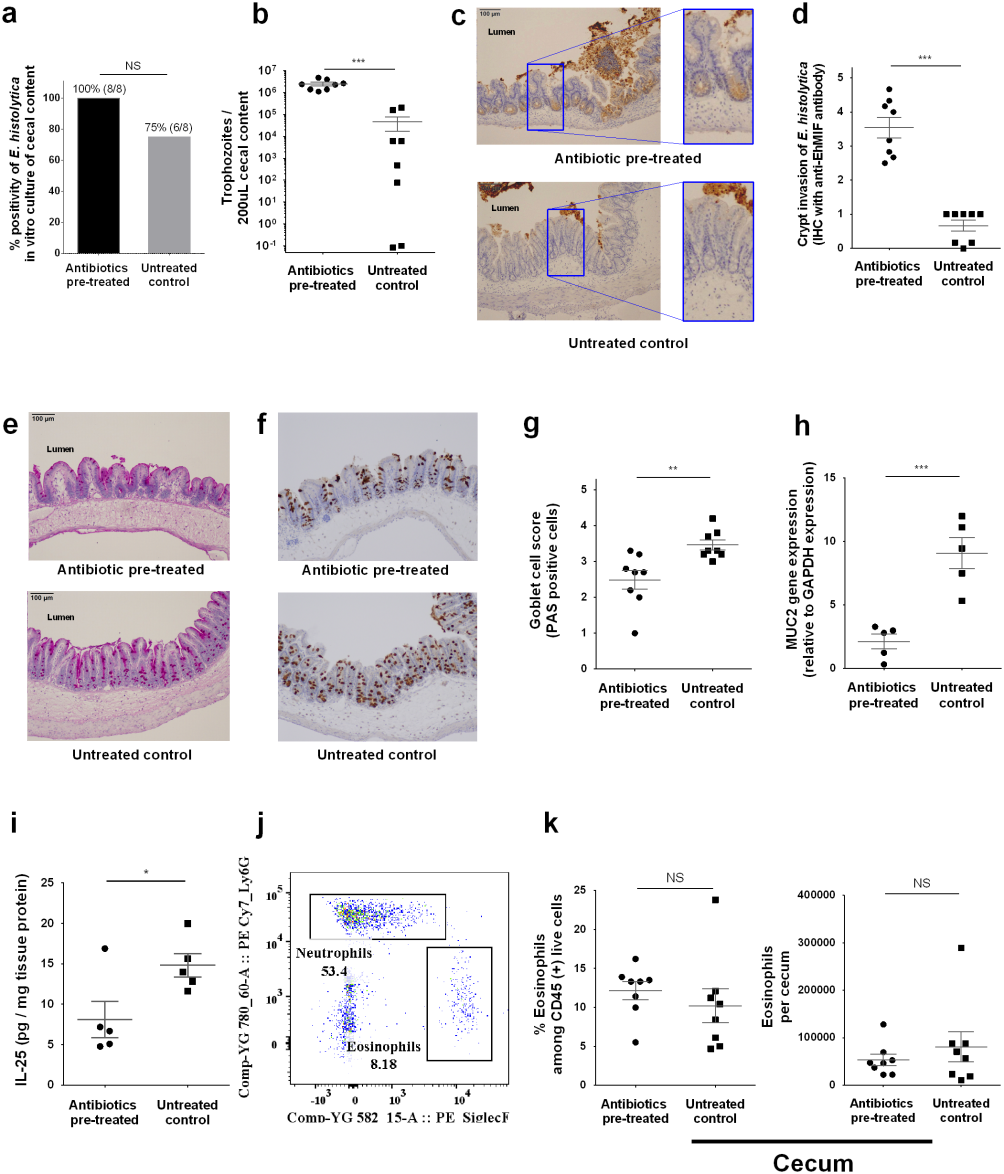
Antibiotic treatment promoted *E*. *histolytica* invasion with disrupted mucosal barrier. Parasite invasion and mucosal barrier disruption upon *E*. *histolytica* challenge were assessed using cecal tissue or cecal content at 24 hours after challenge. **(a, b)** Parasite burden in cecal content was measured by qPCR and *ex vivo* culture positivity was also assessed by putting 200 μL of cecal content to TYI media (n = 8 per group). **(c-g)** *E*. *histolytica* induced epithelial barrier disruption was assessed by immunohistochemistry staining of cecal tissue targeting *E*. *histolytica* derived secreted protein, *E*. *histolytica* migration inhibitory factor (EhMIF) (representative picture in Fig 3c). Mucin containing goblet cells were stained by Periodic acid-Schiff and immunohistochemistry staining of cecal tissue targeting MUC2 (data are presented as representative pictures (e, f). Data are also presented as mean value of histology score blindly scored by three independent observers from similarly conducted two independent experiments, n = 8 per group (d, g)). **(h)** Cecal lysate at 24 hours after *E*. *histolytica* challenge was tested for Muc-2 gene expression by qRT-PCR and is shown normalized to the GAPDH housekeeping gene. **(i)** Cecal cytokine was assessed by lysing 50 mg of cecal sections and quantifying protein via ELISA, and shown normalized to total protein concentration (n = 5 per group (g, h)). **(j,k)** whole cecal tissue was isolated and processed to a single cell suspension and stained for flow cytometry. Eosinophils (CD45^+^ CD11b^+^ SiglecF^+^) were quantified and shown as ratio to CD45^+^ subsets or cell number in whole cecum (n = 8 per group). *P<0.*05*, **P<0.*01*, ***P<0.*001* by Welch’s unequal variance t-test (a, h, i, & k), Mann-Whitney U-test (d & g) or chi-squared test (b). NS, not significant. Error bars represent s.e.m. Eh MIF, *E*. *histolytica* migration inhibitory factor.

In order to assess the tissue invasion of *E*. *histolytica* in cecum at an earlier time point, mice were sacrificed 24 hours after *E*. *histolytica* challenge. While *E*. *histolytica* culture of cecal contents was positive in all antibiotic pre-treated mice and most untreated control mice (Fig 3a), *E*. *histolytica* burden in the cecal lumen was significantly higher in antibiotic pre-treated mice (Fig 3b). Histopathological examination with hematoxylin and eosin stain demonstrated that *E*. *histolytica* trophozoites invaded into mucosa with epithelial cell disruption in antibiotic pre-treated mice, whereas trophozoites were localized within gut lumen in control mice (S2 Fig). Although *E*. *histolytica* could not be identified at crypts or submucosa in the tissue, immunohistochemistry (IHC) using anti-*E*. *histolytica* migration inhibitory factor (anti-EhMIF), which is a secreted protein [22], revealed a higher density of EhMIF at crypts of the cecum in antibiotic pre-treated mice (Fig 3c & 3d), consistent with more severe epithelial damage and invasion by *E*. *histolytica* in antibiotic pre-treated mice. These results strongly suggested that more aggressive *E*. *histolytica* invasion had already occurred within 24 hours after challenge at the site of infection in antibiotic pre-treated mice, although *E*. *histolytica* burden in stool was not different until 4 days after *E*. *histolytica*.

Next, we checked the mucosal barrier upon *E*. *histolytica* challenge. The gut mucosal barrier is the first host defense against intestinal infection by *E*. *histolytica*, as previously shown in MUC-2-deficient mice [23]. Recently, we reported that IL-25 mediated mucosal barrier function plays an important role in the protection from intestinal *E*. *histolytica* infection [24]. Also, it is known that secretion of IL-25 could be reduced by antibiotic induced dysbiosis [25, 26]. At 24 hours after *E*. *histolytica* challenge, we found less mucus-containing MUC2 positive goblet cells by histopathology and less expression of the Muc2 gene (Fig 3e–3h). Also, we confirmed that IL-25 upon *E*. *histolytica* challenge was lower in these mice (Fig 3i). Eosinophils were not different at this time point of infection between antibiotic pre-treated and untreated control mice (Fig 3j & 3k). These results indicate that disturbance of IL-25 and the mucosal barrier were associated with tissue invasion of *E*. *histolytica* in antibiotic pre-treated mice.

### Neutrophil associated gut inflammation was lower in *E*. *histolytica* tissue invasion in antibiotic pre-treated mice

**Fig 4:**
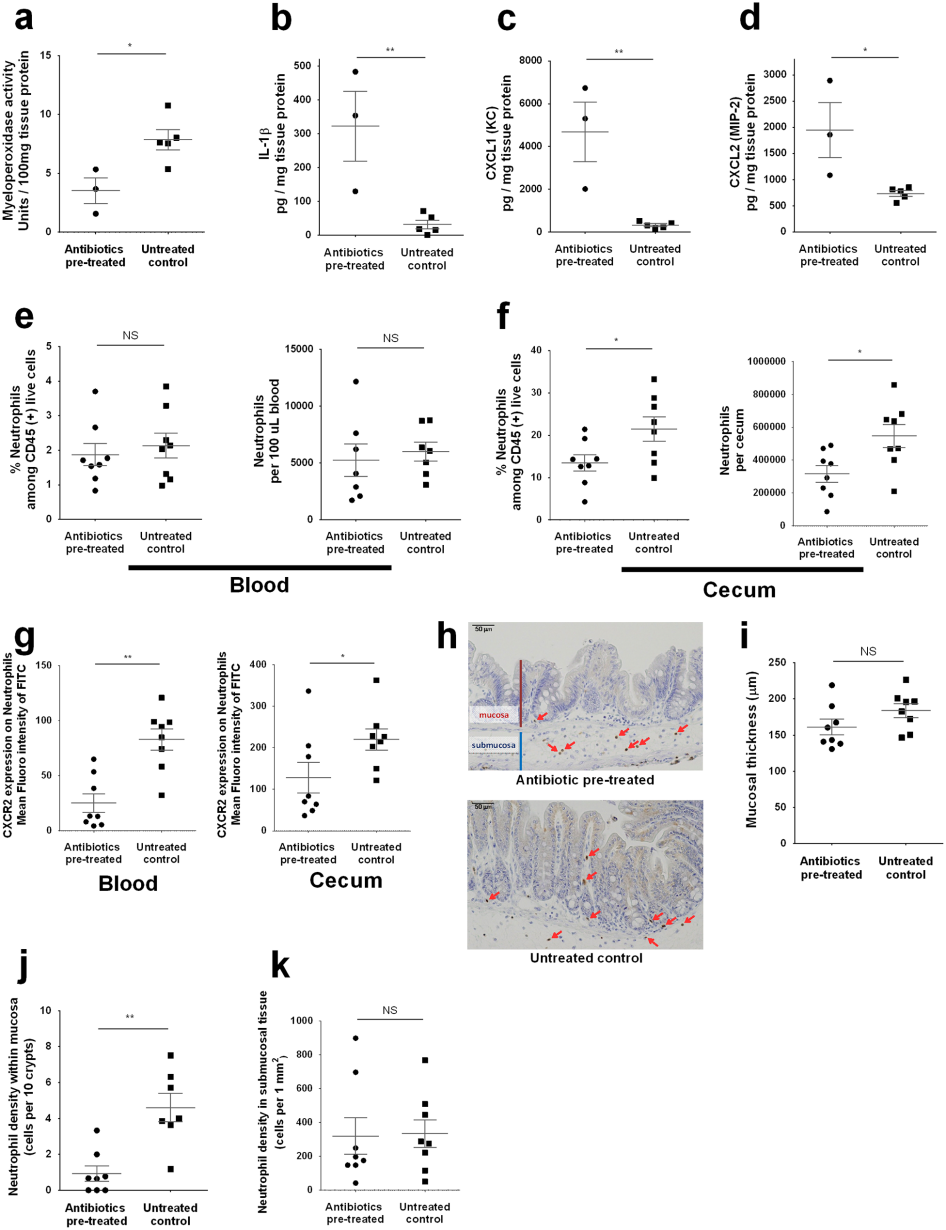
Neutrophil activation and recruitment and CXCR2 expression were decreased in antibiotic pre-treated mice. Neutrophil recruitment to the infection site and its activation upon *E*. *histolytica* challenge were assessed using cecal tissue at 24 hours after challenge. **(a)** Myeloperoxidase activity was assessed by lysing 50 mg cecal lysate in hexadecyltrimethylammonium bromide buffer. Enzyme activity was calculated from standards using recombinant proteins and is shown normalized to total protein concentration (n = 3 or 5 per group). **(b-d)** Cecal cytokines were assessed by lysing 50 mg of cecal sections and quantifying protein via ELISA, and shown normalized to total protein concentration (n = 3 or 5 per group). **(e-g)** Whole cecal tissue was isolated and processed to a single cell suspension and stained for flow cytometry. Neutrophils (CD45^+^ CD11b^+^ Ly6G^high^, representative gating is shown at Fig 3i) were quantified and shown as ratio to CD45^+^ subsets or cell number in whole cecum (n = 8 per each group). **(i-j)** Localization of neutrophils was assessed by immunohistochemistry staining of cecal tissue targeting Ly6G. Mucosal thickness (orange line) was calculated as mean vale of the data from 6 different portions (i), cell number was calculated as mean vale of the data from 3 different portions (data are presented as representative picture (h), or data from n = 8 per each group). *P<0.*05*, **P<0.*01*, ***P<0.*001* by Welch’s unequal variance t-test (a-g, i and k), Mann-Whitney U-test (j) or chi-squared test (b). NS, not significant. Error bars represent s.e.m.

As shown above, despite the more severe tissue damage with delayed clearance of *E*. *histolytica*, stool lipocalin-2 [21], was lower in antibiotic pre-treated mice than those in untreated control mice at early time points of infection (Fig 2f). At later time points, lipocalin-2 was higher in antibiotic-treated and *E*. *histolytica* infected mice, in accordance with pathogen burden (S3a & S3b Fig). These results indicate that neutrophil activation upon *E*. *histolytica* challenge was suppressed early after infection in the antibiotic pre-treated mice, giving a potential explanation for the action of antibiotics in increasing amebiasis severity. Neutrophil myeloperoxidase activity was also measured, and was lower in cecal tissue at 24 hours after *E*. *histolytica* challenge (Fig 4a).

Next, we measured IL-1β level by ELISA, in order to assess the recognition of *E*. *histolytica* by the intestinal epithelial cells and innate immune system. IL-1β is a pro-inflammatory cytokine, and it has been shown that NLRP3-inflammasome caspase-1 mediated IL-1β is secreted from a macrophage cell line upon contact with *E*. *histolytica* [27, 28]. It was also reported that NLRP3-inflammasome activation could be disturbed by antibiotic induced dysbiosis, resulting in more severe airway infection by influenza virus [29]. Opposed to our hypothesis, however, IL-1β as well as the neutrophil chemoattractant chemokines CXCL1 and CXCL2 at 24 hours after *E*. *histolytica* challenge were significantly higher in antibiotic pre-treated mice compared to those in untreated control mice (Fig 4b–4d) although they were elevated in response to *E*. *histolytica* challenge compared to baseline levels in both antibiotic pre-treated and untreated control mice (S4a–S4c Fig). Considered together, disturbed neutrophil activation upon *E*. *histolytica* challenge in antibiotic pre-treated mice was not caused by impaired recognition of *E*. *histolytica* as manifest by IL-1β or chemokines, leading us to ask if it might be caused by decreased neutrophil responses to these chemokines.

### Neutrophil number in the gut and expression of CXCR2 were lower upon *E*. *histolytica* infection in antibiotic pretreated mice

As presented above, neutrophil activation was suppressed despite tissue invasion by *E*. *histolytica* in antibiotic treated mice. These mice also paradoxically expressed higher neutrophil chemoattractant chemokines. We hypothesized that inhibition of CXCR2 expression on neutrophils, which is the main receptor for the chemokines CXCL1 and CXCL2, could explain the relative lack of gut neutrophil abundance. In order to assess this hypothesis, we checked the number and expression of surface markers of the Ly6G high neutrophil population (Fig 3j) in the blood and cecal lamina propria of mice at 24 hours after *E*. *histolytica* challenge by flow cytometry. As expected, gut but not systemic neutrophil numbers were depressed in antibiotic treated mice (Fig 4e & 4f). In addition, CXCR2 expression on neutrophils was lower both in blood and cecum (Fig 4g), whereas expression of the other surface molecules were either not different or higher in antibiotic pre-treated mice (S5c & S5d Fig). Next, cecal tissue at 24 hours after challenge was stained by anti-Ly6G antibody in order to assess neutrophil localization in cecal tissue (Fig 4h). We confirmed mucosal thickness was not different between 2 groups (Fig 4i), then compared the frequency of Ly6G positive neutrophils in the mucosa (cells per 10 crypts) and submucosal tissue (cells per unit area). Neutrophils in the mucosa were lower in antibiotic pre-treated mice (Fig 4j) whereas neutrophil number in submucosal tissue was not different between the 2 groups (Fig 4k), suggesting that efficient neutrophil migration from submucosal tissue to the site of infection, which plays an important role in protection from tissue invasion of microorganisms [30, 31], was disturbed in antibiotic pre-treated mice. These results indicate that lower expression of CXCR2 on neutrophils was a potential cause of impaired neutrophil recruitment to the infection site, which resulted in a susceptible phenotype to *E*. *histolytica* challenge in the antibiotic pre-treated mice.

### Impact of CXCR2 on disease severity

**Fig 5:**
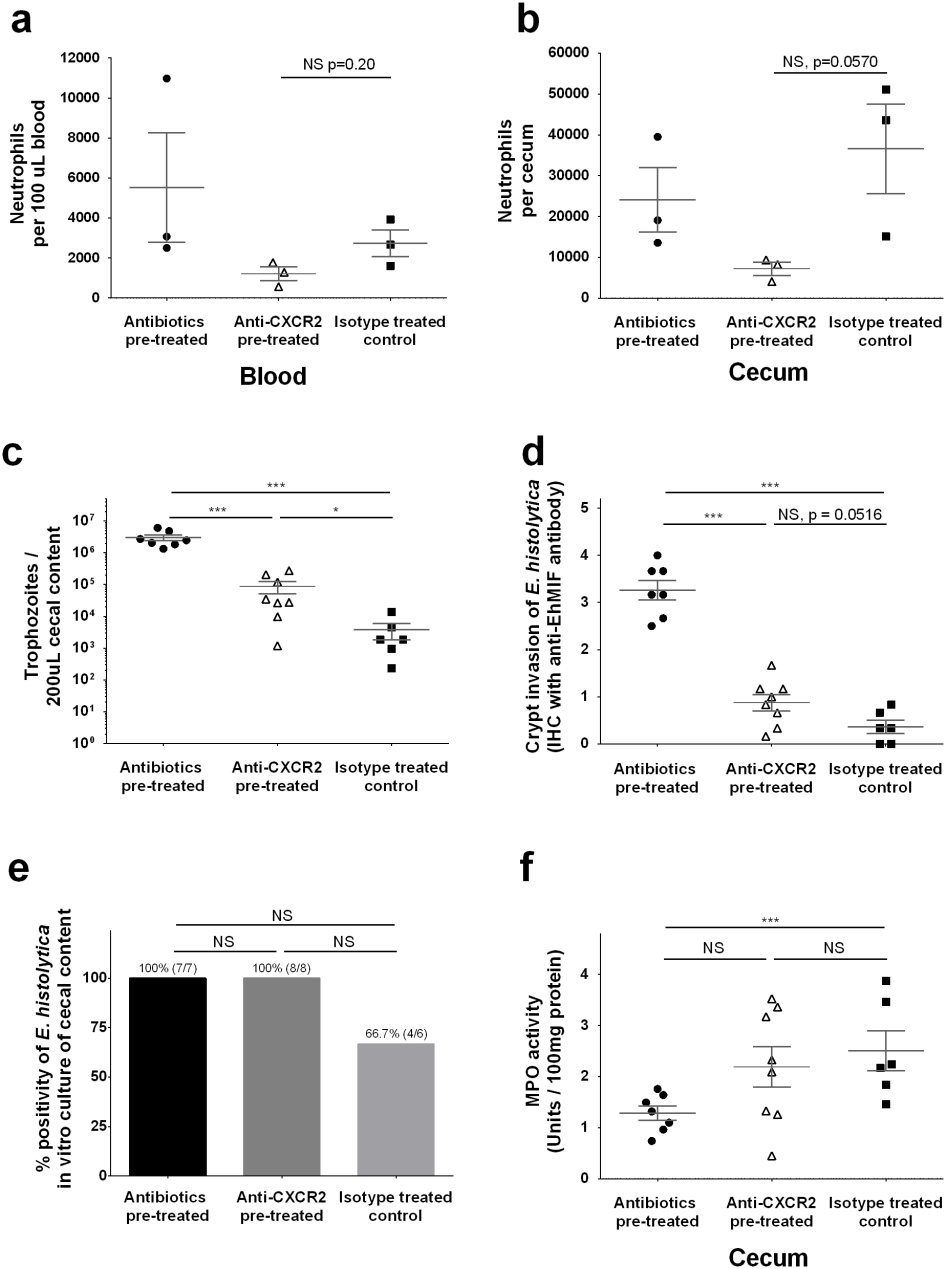
Anti-CXCR2 antibody treatment increased susceptibility of non-antibiotic treated mice to *E*. *histolytica*. Rat anti-mouse CXCR2 monoclonal antibody was administered 2 hours prior to *E*. *histolytica* challenge to blockade CXCR2. **(a, b)** Whole cecal tissue or 100 μL peripheral blood was collected and processed to a single cell suspension and stained for flow cytometry. Neutrophils (CD45^+^ CD11b^+^ Ly6G^high^, representative gating is shown at Fig 3i) were shown as cell number in whole cecum (data are representative of similarly conducted two independent experiments, n = 3 per each group). **(c)** Myeloperoxidase activity was assessed by lysing 50 mg of cecum in hexadecyltrimethylammonium bromide buffer. Enzyme activity was calculated from standards using recombinant proteins and is shown normalized to total protein concentration. **(d)** Parasite burden in cecal content was measured by qPCR using 200 μL of cecal content. **(e)** *E*. *histolytica* induced epithelial barrier disruption was assessed by immunohistochemistry staining of cecal tissue targeting *E*. *histolytica* derived secreted protein, *E*. *histolytica* migration inhibitory factor (EhMIF) (data are presented as mean value of histology score blindly scored by three independent observers). **(f)** *Ex vivo* culture positivity was also assessed by placing 200 μL of cecal content in TYI media. *P<0.*05*, **P<0.*01*, ***P<0.*001* by one-way ANOVA-test (a-e) or chi-squared test (d). NS, not significant. Error bars represent s.e.m. MPO, myeloperoxidase; EhMIF, *E*. *histolytica* migration inhibitory factor.

In order to assess the impact of chemokine mediated neutrophil recruitment via CXCR2 on *E*. *histolytica* infection, we tested if blocking of CXCR2 would render control (non antibiotic treated) mice susceptible to *E*. *histolytica*. CXCR2 was neutralized using a monoclonal antibody (rat anti-mouse CXCR2 IgG2A) injected intraperitoneally 2 hours before *E*. *histolytica* challenge [32]. Neutrophil number after *E*. *histolytica* challenge in cecum, but not in peripheral blood, was suppressed in anti-CXCR2 pre-treated mice compared to isotype control treated mice, although both are not statistically significant (Fig 5a & 5b). *E*. *histolytica* burden at 24 hours after challenge was significantly higher in anti-CXCR2 pre-treated mice compared to isotype control treated mice, although lower than in the antibiotic pre-treated mice (Fig 5c). Also, tissue invasion of *E*. *histolytica* was more severe (Fig 5d), and *E*. *histolytica* culture from cecal contents was positive in all anti-CXCR2 pre-treated mice as seen in antibiotic pre-treated mice (Fig 5e). Interestingly, the impact of CXCR2 blocking on tissue MPO activity (Fig 5f) as well as *E*. *histolytica* burden and histopathological score was less profound than that seen by antibiotic pre-treatment, suggesting that the low expression of CXCR2 was not the sole explanation for the higher susceptibility of antibiotic pre-treated mice to *E*. *histolytica* challenge.

## Discussion

**Fig 6:**
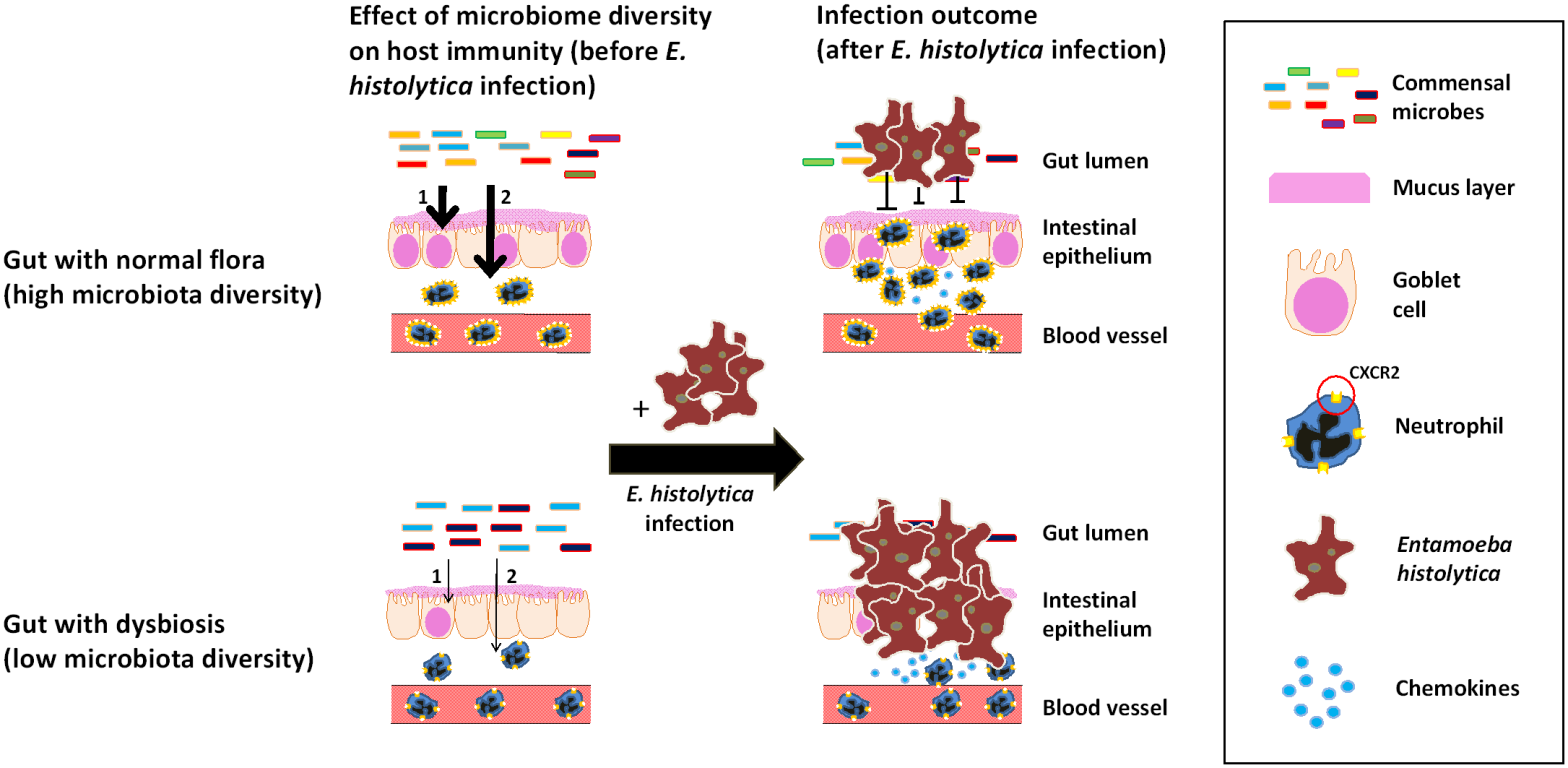
Graphical summary: Effects of microbiota on immune response to *E*. *histolytica* infection. During dysbiostic state (bottom figures), diversity of gut commensal microbes is low compared to healthy state (upper figures). IL-25 mediated mucosal protection by epithelial cells (1) and CXCR2 expression on neutrophils (2) upon *E*. *histolytica* challenge are diminished in dysbiostic state, allowing more severe tissue invasion by *E*. *histolytica*.

The most important finding of this study is that gut microbiome dysbiosis increases susceptibility to amebic colitis in humans and in the mouse model, and that one mechanism of this increased susceptibility is downregulated neutrophil recruitment to the gut. In children who developed symptomatic colitis by *E*. *histolytica*, the gut microbiota had lower diversity than those in children who showed colonization. Moreover, we found that children developing amebic colitis had lower microbiome diversity in the month preceding amebic colitis than those without *E*. *histolytica* infection, although these data came from 2 different cohort studies at the same location. We extended these studies in the mouse model of amebic colitis, demonstrating that mechanisms of dysbiosis-mediated increased susceptibility were downregulation of neutrophil CXCR2 and attendant failure of neutrophil recruitment to the site of infection. Although changes in gut microbiota can induce or conversely reduce gut inflammation, dysbiosis (represented by lower microbiota diversity) in this case reduced the neutrophil activation in the gut, allowing more severe intestinal damage by *E*. *histolytica* (Fig 6).

Antibiotic use is pervasive in children in low and middle income countries and a likely cause of dysbiosis [33]. In fact, we found that antibiotics were frequently used in participants of PROVIDE study in Bangladesh with an average of 25.5 new prescriptions over first 2 years of life, although such data was not available in NIH birth cohort. It is also known in mouse studies that the gut microbiome is essential not only for the development of the immune system but also for maintenance of homeostasis [34, 35]. In the present study, we sought to mimic our cohort observation in mice by administration of an antibiotic cocktail (ampicillin, neomycin, vancomycin and metronidazole) [29, 36–40]. Strikingly, C57BL/6 mice, which are naturally resistant to *E*. *histolytica* infection [41], had increased susceptibility after antibiotic induced dysbiosis. We confirmed that mucus secretion was disturbed with decreased IL-25 level at the site of infection in antibiotic pre-treated mice, which might allow more severe tissue invasion of *E*. *histolytica* [42]. Interestingly, neutrophil number and activation at the site of infection were suppressed as assessed by fecal lipocalin-2, myeloperoxidase activity and neutrophil number despite more tissue damage by *E*. *histolytica* in antibiotic pre-treated mice. We concluded that insufficient neutrophil recruitment as well as suppressed mucus secretion at the site of infection allowed *E*. *histolytica* tissue invasion at 24 hours after infection (Fig 3), which were followed by more aggressive colitis with more severe weight loss and delayed clearance of *E*. *histolytica* from stool (Fig 2).

Upon intestinal damage by infection, DAMPs and PAMPs from damaged tissue and pathogen respectively are recognized by intestinal epithelial cells and antigen presenting cells (APCs). This is followed by the production of neutrophil chemoattractant chemokines including CXCL1 and CXCL2 mainly produced by activated tissue residential macrophages [43], which in turn induce neutrophil recruitment to the site of infection [44]. However, CXCL1 and CXCL2 as well as IL-1β upon *E*. *histolytica* infection were significantly higher in antibiotic pre-treated mice compared to untreated control mice, indicating that increased susceptibility to infection was not due to poor recognition of PAMPs nor DAMPs [29] but instead due to failure of neutrophil response to chemokines.

We checked the expression of CXCR2, which is the main chemokine receptor on neutrophils for CXCL1 and CXCL2 [45], because expression of CXCR2 is critically important for neutrophil associated protection against various infectious diseases [32, 46–48]. We found that CXCR2 expression on neutrophils both in blood and cecum after *E*. *histolytica* challenge was lower in antibiotic pre-treated mice than that in untreated control mice. Finally, we confirmed that anti-CXCR2 pre-treated mice showed significantly higher *E*. *histolytica* burden than untreated control mice at 24 hours after *E*. *histolytica* challenge. From these results, we concluded that down-regulation of CXCR2 is an important but not sole mechanism by which dysbiosis diminished neutrophil mediated protection. Furthermore, our results indicate that dysbiosis might affect the susceptibility to infection by other organisms or inflammatory bowel disease [32, 46–48] by altering neutrophil recruitment or function, by suppressing CXCR2 expression [49]. On the other hand, our results was contradict with the previous paper reported by Houpt et al. which demonstrated that C57BL/6 mice remained resistant to *E*. *histolytica* infection after depletion of neutrophils [14]. Although this might be explained by the fact that Houpt et al. did not disrupt the microbiome with antibiotics prior to infection of C57BL/6 mice with antibiotics, further investigations for elucidating the impact of microbiota on neutrophil function, including not only recruitment of neutrophils but also amebicidal activity, will be needed in future study.

There are some limitations to be considered. First, our results in Bangladeshi children have not been generalized to children in other countries, although they do represent two different cohorts (PROVIDE study and NIH birth cohort) performed at the same district (Mirpur, Dhaka, Bangladesh). Second, we created dysbiosis in the murine model by administering a broad spectrum antibiotic cocktail. However, the composition of the gut microbiome is different between human and mice [50]. The bacterial species which influence disease severity of amebiasis are therefore yet to be fully identified although one paper has shown the relationship between the presence of *Prevotella copri* and disease severity of amebic colitis [7]. A third limitation is that we do not understand the mechanism of microbiota-mediated changes in CXCR2 expression on neutrophils upon *E*. *histolytica* infection. Fourth we have not assessed amebicidal activities of neutrophils although our results suggested that they are suppressed during antibiotic induced dysbiosis (Fig 5). In the future the colonization of mice by specific bacteria [8, 51] and assessing amebicidal activity of their neutrophils [17] may be used to identify the bacteria responsible for resistance and to elucidate mechanisms of protection. Finally, we could not assess neutrophil CXCR2 in the bloodstream of the children with *E*. *histolytica* infection to test the extent that it was downregulated as in the mouse model. Future work should assess dynamically the impact of antibiotic use on neutrophil function during amebic infection in a human cohort.

In conclusion, we demonstrated that antibiotic-induced dysbiosis mediated susceptibility to amebic colitis through decreased neutrophil recruitment to the gut, at least partially due to decreased surface expression of neutrophil CXCR2. Demonstration that antibiotics can impair neutrophil chemotaxis to a site of infection may be of broader importance than just amebiasis for the insight that it provides into gut microbiome regulation of the immune system.

## Methods

### Ethics statements

Stool samples and the clinical information from children who developed amebic colitis came from Performance of Rotavirus and Polio Vaccines in Developing Countries (PROVIDE) study, whereas those from children who showed *E*. *histolytica* colonization were collected in NIH birth cohort. Details of the PROVIDE study and NIH birth cohort were described previously [19, 20]. In brief, the PROVIDE study was performed at a peri-urban slum of Mirpur in Dhaka and included 700 infants enrolled at the first week of their age, and followed up at least until 53 weeks (2 year) of age. NIH birth cohort is also prospective cohort study of enteric infections in infants from the same study site as PROVIDE. In both studies, written informed consents were provided by parents or guardian on behalf of all infant participants at enrollment. Field workers visited the child’s household twice weekly to capture any diarrhea event and collected stool samples from these as well as a monthly surveillance stool sample. The diagnosis of amebic colitis was confirmed by qPCR assay [7]. The protocol and informed consent (English and Bangla) and all amendments were reviewed and approved by the Research Review Committee (RRC) and Ethics Review Committee at the International Centre for Diarrhoeal Disease Research, Bangladesh (icddr, b) and the Institutional Review Board of the University of Virginia prior to implementation. Trial registration: ClinicalTrials.gov NCT01375647 for PROVIDE study and NCT02764918 for NIH birth cohort.

All animal experiments conducted in this study were carried out in accordance with the Animal Welfare Act and the recommendations in the Guide for the Care and Use of Laboratory Animals of the National Institutes of Health. All procedures were approved by the Institutional Animal Care and Use Committee of the University of Virginia (Protocol Number: #4126).

### Mice

Female 6-week-old C57BL/6 mice (Jackson Laboratories, Charles River) were housed in a specific-pathogen-free facility in micro-isolator cages and provided autoclaved food (Lab Diet 5010) and water.

### Antibiotic pre-treatment for dysbiosis

Mice were treated with an antibiotic cocktail, consisting of 1.0 gram per liter of ampicillin (Sigma), neomycin (Sigma) and metronidazole (Sigma) and 0.5 gram per liter of vancomycin (Hopsra) in drinking water, as previously used to induce dysbiosis [29, 36–40]. All antibiotics except metronidazole were continued until harvest, with metronidazole discontinued 72 hours prior to *E*. *histolytica* challenge due to potential amebicidal activity.

### Blocking of CXCR2 by monoclonal antibody

Twenty microgram of rat monoclonal anti-mouse CXCR2 antibody (clone # 242216, R&D) or 20 μg of rat IgG2A isotype (R&D) control was injected into the peritoneal cavity 2 h before infection [32, 52].

### *E*. *histolytica* culture and intracecal injection

Trophozoites for intracecal injections were originally derived from laboratory strain HM1:IMSS (American Type Culture Collection) that have been sequentially passaged in vivo through mouse cecum. Cecal contents of infected mice were cultured in complete trypsin-yeast-iron (TYI-33) medium supplemented with Diamond vitamin mix (JRH Biosciences), 100 U/ml of penicillin and 100 μg/ml of streptomycin, and bovine serum (Sigma-Aldrich) [41, 53]. Prior to injection, trophozoites were grown to log phase, and 2 x 10^6^ parasites were suspended in 150 μl culture medium and injected intracecally [41].

### Assessment of clinical symptoms

Body weight and clinical score were monitored weekly before *E*. *histolytica* challenge and every 24 hours after challenge. Clinical score was calculated as the total score of 6 different variables [54] (S1 Table). DNA was extracted from 50 mg of stool as described later. For ELISA, 50 mg of stool was reconstituted in 1 mL of PBS containing 0.1% Tween 20 (100 mg/ml) and vortexed for 20 min to get a homogenous fecal suspension. These samples were then centrifuged for 10 min at 12,000 rpm at 4°C. Clear supernatants were collected and stored at -20°C until analysis. Lipocalin-2 levels were estimated in the supernatants using Duoset murine Lcn-2 ELISA kit (R&D Systems) [21, 55]. Additionally, inflammation-associated rectal bleeding was assessed by examination of blood in the stool by Hemooccult II SENSA (Beckman Coulter) [56].

### *E*. *histolytica* infection rate at sacrifice

Two hundred microliter of cecal contents were collected at sacrifice of the infected mice, and cultured in complete TYI-S-33 medium with supplemental antibiotics for 3 days at 37°C. *E*. *histolytica* infection was confirmed by the direct microscopic examination of cultured tube at 72 hours. Infection rate was presented by the ratio of infected mice among total number of mice challenged by *E*. *histolytica*.

### Histopathology and scoring

After fixation for 24 hours in Bouin’s solution, mouse cecal tissue was washed and stored in 70% ethanol. Paraffin embedding, H&E and PAS staining were processed by the University of Virginia Research Histology core. Immunochemistry (IHC) staining was performed by the University of Virginia Biorepository and Tissue Research Facility. IHC staining was performed using the Dako Autostainer Universal System with a primary antibody directed against *E*. *histolytica* macrophage migration inhibitory factor [22], rabbit anti-MUC2 polyclonal antibody (Cat. No. PA5-21329) or rat anti-mouse Ly6G (clone No. 1A8, Biolegend). Scoring was based on intensity and abundance of EhMIF staining in mucosa by crypt invasion of *E*. *histolytica*, and based on abundance of mucin containing cells for goblet cell score (staining scale was between 0 and 5). Histopathological scoring was done by three independent blinded scorers.

### Myeloperoxidase activity in cecum

Myeloperoxidase (MPO) activity was determined as previously described [23, 57]. In brief, cecal tissues were homogenized in hexadecyltrimethylammonium bromide buffer. Samples were centrifuged at 10,000 g (10 minutes, 4 degree), and the supernatant was collected, and total protein concentration was assessed by a BCA assay according to the manufacturer’s instructions (Pierce). Two hundred μl of 0.53 μmol/L o-dianisidine dihydrochloride with 1% hydrogen peroxide were added to 7 uL of supernatant and the absorbance was determined at 450 nm. Results were normalized to total protein concentration.

### Quantification of tissue protein

Resected cecal tissue was bead beaten for 1 min in 250 μl of lysis buffer I (1× HALT protease inhibitor (Pierce), 5 mM HEPES). Lysis buffer II (250 μl) was added (1× HALT protease inhibitor, 5 mM HEPES, 2% Triton X-100 (Sigma-Aldrich)) and the tubes were inverted gently. Tissue samples were incubated on ice for 30 min, followed by a 5 min spin at 13,000 x *g* at 4°C. The supernatant was removed to a fresh tube, and total protein concentration was assessed by a BCA assay according to the manufacturer’s instructions (Pierce). IL-1β was measured using Ready-Set-Go! ELISA kit (eBioscience). CXCL1, CXCL2 and IL-25 were measured using R&D Systems Duoset ELISA kits. All procedures are performed according to the manufacturers’ instructions. All data were expressed relative to total protein concentration.

### Quantitative real-time PCR

For DNA extraction from stool (50 mg) or cecal contents (200 μL), QIAamp Fast DNA Stool Mini Kit (Qiagen) was used. All samples were bead beaten for 2 min prior to DNA extraction. All other procedures were performed according to the manufacturer’s instruction. For RNA purification, RNeasy isolation kit (Qiagen) was applied for 50 mg of cecal tissue sample. RNA was reverse transcribed with the Tetro cDNA synthesis kit (Bioline). All procedures were performed according to the manufacturer’s instructions. For the quantification of *E*. *histolytica*, a standard curve was prepared from trophozoites, and quantitative PCR (qPCR) targeting small subunit ribosomal RNA gene [58] was utilized. Probe, primers and annealing temperature (AT) were as follows: Eh-probe: Fam/TCATTGAATGAATTGGCCATTT/BHQ; Eh-forward: ATTGTCGTGGCATCCTAACTCA; Eh-reverse: GCGGACGGCTCATTATAACA, AT: 62.4°C. MUC2 gene expression was quantified by qPCR using Sensifast SYBR & Fluorescein Mix (Bioline). Gene expression was normalized to the GAPDH gene expression. Primer sequences and annealing temperature (AT) used in this study were as follows; MUC2 gene (forward: 5’- GCTGACGAGTGGTTGGTGAATG - 3’; reverse: 5’ - GATGAGGTGGCAGACAGGAGAC - 3’; AT: 60.0°C) and GAPDH (forward: 5’ -AAC TTT GGC ATT GTG GAA GG - 3’; reverse: 5’ -ACA CAT TGG GGG TAG GAA CA – 3’; AT: 62.4°C).

### Microbiome diversity analysis from human stool samples

DNA was extracted from fecal material using a modified QiaAmp stool DNA extraction protocol which incorporates a 3 min “bead-beating” step as per standard study protocols [59]. Human DNA was removed from *E*. *histolytica* positive samples using a Microbiome DNA Enrichment Kit used by the manufacturer’s direction (NEB). The 255bp V4 region was completely sequenced in both forward and reverse orientation using the Miseq V3 kit (also used by the manufacturer’s direction). The sequencing library was prepared using phased Illumina-eubacteria primers to both amplify the V4 16S region rDNA (515–806), add the adaptors necessary for illumina sequencing and the GOLAY index necessary for de-multiplexing after parallel sequencing [60, 61]. As a positive control, DNA extracted from the HM-782D Mock Bacteria Community (ATCC through BEI Resources) was added, and as a control for reagent and laboratory contamination a no-template control reaction was added. Sequencing, quality control and OTU assignation using the QIIME pipeline was performed by the Institute for Genome Sciences Core facility (Baltimore). The data was then visualized and Shannon Diversity Scores determined using the Seed Program [62]. Stool samples were collected from PROVIDE study children.

### Flow cytometry analyses

Single cell suspensions from cecal lamina propria were prepared as previously described [54, 63]. Briefly the tissue was thoroughly rinsed in Hank’s balanced salt solution (HBSS) supplemented with 5% FBS. Epithelial cells were removed by gentle shaking for 40 min at 37°C in HBSS with 15 mM HEPES, 5 mM EDTA, 10% FBS and 1 mM dithiothreitol. Halfway through the incubation, cecal tissue samples were transferred to fresh buffer. Next, cecal tissue samples were thoroughly chopped using scissors and digested in RPMI 1640 containing 0.17 mg ml^–1^ Liberase TL (Roche) and 30 μg ml^–1^ DNase (Sigma). Samples were digested for 30 min at 37°C with shaking. Samples were then spun down at 300 x *g* and resuspended in HBSS with 5% FBS and 25 mM HEPES before passage through a 100 μm cell strainer followed by a 40 μm cell strainer (both Fisher Scientific). Cells were counted and the density adjusted to 5 × 10^6^ cells per ml. Cell suspensions (200 μl) were aliquoted into each well of a 96-well plate for antibody staining. For staining, cells were initially blocked with TruStain fcX (anti-mouse CD16/32 antibody, BioLegend) for 15 min at room temperature. Cells were spun down and resuspended in LIVE/DEAD Fixable Aqua (Life Technologies) for 30 min in the dark place at room temperature. Cells were washed twice and stained with fluorochrome conjugated antibodies. Flow cytometry was performed on an LSR Fortessa cytometer (BD Biosciences) and all data analysis was performed via FlowJo (Tree Star). Data was analyzed as the ratio to CD45 positive cells or cell counts calculated by counting beads (Molecular Probes). Fluorescent conjugated antibodies used for flow cytometry are shown in S2 Table.

### Statistical analysis

ANOVA was used for differences among multiple groups. Welch’s unequal variance t-test, Mann-Whitney U-test or chi-squared test were used as appropriate for comparing valuables in 2 groups. A p value below 0.05 was considered significant. All statistical tests were done using GraphPad Prism software.

## Supporting information

S1 Fig Diversity of microbiome was decreased prior to amebic colitis. The Shannon diversity index was examined using stool samples collected from children followed longitudinally in an urban slum in Dhaka, Bangladesh who developed amebic colitis within the first 2 years of life (n = 18). The Shannon diversity index was measured one month prior to amebic colitis, and compared to that from children who did not develop *E*. *histolytica* infection (n = 72). *P<0.*05*, by Welch’s unequal variance t-test. Error bars represent standard error of the mean (s.e.m).
(PDF)

S2 Fig Tissue invasion into intestinal epithelial cells by *E*. *histolytica* in antibiotic pre-treated mice but not in untreated control mice. Histopathological examinations by hematoxilin and eosin stain were performed using cecal tissue collected from mice sacrificed at 24 hours after *E*. *histolytica* challenge. Yellow arrows represent *E*. *histolytica*. **(a, b)** Representative pictures of tissue invasion by *E*. *histolytica* in antibiotic pretreated mice. **(c, d)** Representative pictures of ulcerative lesions in untreated control mice. **(a, c)** x200 represents yellow boxes in a and d, **(b, d)** x400 represents yellow boxes in a and c.
(PDF)

S3 Fig Immune responses at day 9 are correlated with infection outcome of *E*. *histolytica*. Antibiotic pre-treated or untreated control wild type C57BL/6 mice were infected with 2 x 10^6^ *E*. *histolytica* trophozoites intracecally, and were sacrificed at day 9. **(a)** *E*. *histoltyica* burden was measured by qPCR. **(b, c)** lipocalin-2 and anti-lectin IgA were assessed by ELISA using 200 μL of cecal contents. Data are representative from similarly conducted two independent experiments. *P<0.*05*, **P<0.*01*, ***P<0.*001* by Welch’s unequal variance. NS, not significant. Error bars represent s.e.m.
(PDF)

S4 Fig IL-1β and neutrophil attractant chemokines at baseline. Antibiotic pre-treated or untreated control wild type C57BL/6 mice were sacrificed at 2 weeks of antibiotics in order to see the baseline data of IL-1β, CXCL1 and CXCL2 in cecal tissue before *E*. *histolytica* challenge. Cecal cytokines were assessed by lysing 50mg of cecal sections and quantifying protein via ELISA, and shown normalized to total protein concentration (data from single experiment, n = 5 per group). *P<0.*05*, **P<0.*01*, ***P<0.*001* by Welch’s unequal variance. NS, not significant. Error bars represent s.e.m.
(PDF)

S5 Fig Surface expression of molecules on neutrophils. Antibiotic pre-treated or untreated control wild type C57BL/6 mice were infected with 2 x 10^6^ *E*. *histolytica* trophozoites intracecally. Surface protein expression levels were assessed as mean fluorescence intensity (MFI) by flow cytometry using single cell suspension from blood and lamina propria. **(a, b)** Surface protein expression levels before *E*. *histolytica* challenge. **(c, d)** Surface protein expression levels at 24 hours after *E*. *histolytica challenge*. Data are from a single experiment, n = 8 per group. *P<0.*05*, **P<0.*01*, ***P<0.*001* by Welch’s unequal variance t-test NS, not significant. Error bars represent s.e.m.
(PDF)

S1 Table Clinical scoring. (PDF)

S2 Table Conjugated antibodies used flowcytometry. (PDF)

## References

[1] Lozano R, Naghavi M, Foreman K, Lim S, Shibuya K, Aboyans V, et al Global and regional mortality from 235 causes of death for 20 age groups in 1990 and 2010: a systematic analysis for the Global Burden of Disease Study 2010. Lancet. 2012;380(9859):2095–128. doi: 10.1016/S0140-6736(12)61728-0 . 23245604 10.1016/S0140-6736(12)61728-0 PMC10790329

[2] Haque R, Huston CD, Hughes M, Houpt E, Petri WA Jr. Amebiasis. N Engl J Med. 2003;348(16):1565–73. doi: 10.1056/NEJMra022710 . 12700377 10.1056/NEJMra022710

[3] Gathiram V, Jackson TF. A longitudinal study of asymptomatic carriers of pathogenic zymodemes of Entamoeba histolytica. South African medical journal = Suid-Afrikaanse tydskrif vir geneeskunde. 1987;72(10):669–72. . 2891197

[4] Haque R, Ali IM, Petri WA Jr. Prevalence and immune response to Entamoeba histolytica infection in preschool children in Bangladesh. The American journal of tropical medicine and hygiene. 1999;60(6):1031–4. . 10403338 10.4269/ajtmh.1999.60.1031

[5] Watanabe K, Aoki T, Nagata N, Tanuma J, Kikuchi Y, Oka S, et al Clinical significance of high anti-entamoeba histolytica antibody titer in asymptomatic HIV-1-infected individuals. The Journal of infectious diseases. 2014;209(11):1801–7. doi: 10.1093/infdis/jit815 . 24338349 10.1093/infdis/jit815

[6] Irusen EM, Jackson TF, Simjee AE. Asymptomatic intestinal colonization by pathogenic Entamoeba histolytica in amebic liver abscess: prevalence, response to therapy, and pathogenic potential. Clinical infectious diseases: an official publication of the Infectious Diseases Society of America. 1992;14(4):889–93. . 1576284 10.1093/clinids/14.4.889

[7] Gilchrist CA, Petri SE, Schneider BN, Reichman DJ, Jiang N, Begum S, et al Role of the Gut Microbiota of Children in Diarrhea Due to the Protozoan Parasite Entamoeba histolytica. The Journal of infectious diseases. 2016;213(10):1579–85. doi: 10.1093/infdis/jiv772 . 26712950 10.1093/infdis/jiv772 PMC4837909

[8] Burgess SL, Buonomo E, Carey M, Cowardin C, Naylor C, Noor Z, et al Bone marrow dendritic cells from mice with an altered microbiota provide interleukin 17A-dependent protection against Entamoeba histolytica colitis. mBio. 2014;5(6):e01817 . 25370489 10.1128/mBio.01817-14 PMC4222101

[9] Burgess SL, Saleh M, Cowardin CA, Buonomo E, Noor Z, Watanabe K, et al Role of Serum Amyloid A, Granulocyte-Macrophage Colony-Stimulating Factor, and Bone Marrow Granulocyte-Monocyte Precursor Expansion in Segmented Filamentous Bacterium-Mediated Protection from Entamoeba histolytica. Infection and immunity. 2016;84(10):2824–32. doi: 10.1128/IAI.00316-16 . 27456830 10.1128/IAI.00316-16 PMC5038085

[10] Seydel KB, Stanley SL Jr. Entamoeba histolytica induces host cell death in amebic liver abscess by a non-Fas-dependent, non-tumor necrosis factor alpha-dependent pathway of apoptosis. Infection and immunity. 1998;66(6):2980–3. . 9596776 10.1128/iai.66.6.2980-2983.1998 PMC108298

[11] Velazquez C, Shibayama-Salas M, Aguirre-Garcia J, Tsutsumi V, Calderon J. Role of neutrophils in innate resistance to Entamoeba histolytica liver infection in mice. Parasite immunology. 1998;20(6):255–62. . 9651927 10.1046/j.1365-3024.1998.00128.x

[12] Seydel KB, Zhang T, Stanley SL Jr. Neutrophils play a critical role in early resistance to amebic liver abscesses in severe combined immunodeficient mice. Infection and immunity. 1997;65(9):3951–3. . 9284178 10.1128/iai.65.9.3951-3953.1997 PMC175565

[13] Jarillo-Luna RA, Campos-Rodriguez R, Tsutsumi V. Entamoeba histolytica: immunohistochemical study of hepatic amoebiasis in mouse. Neutrophils and nitric oxide as possible factors of resistance. Experimental parasitology. 2002;101(1):40–56. . 12243737 10.1016/s0014-4894(02)00021-8

[14] Asgharpour A, Gilchrist C, Baba D, Hamano S, Houpt E. Resistance to intestinal Entamoeba histolytica infection is conferred by innate immunity and Gr-1+ cells. Infection and immunity. 2005;73(8):4522–9. . 16040963 10.1128/IAI.73.8.4522-4529.2005 PMC1201199

[15] Estrada-Figueroa LA, Ramirez-Jimenez Y, Osorio-Trujillo C, Shibayama M, Navarro-Garcia F, Garcia-Tovar C, et al Absence of CD38 delays arrival of neutrophils to the liver and innate immune response development during hepatic amoebiasis by Entamoeba histolytica. Parasite immunology. 2011;33(12):661–8. doi: 10.1111/j.1365-3024.2011.01333.x . 21919917 10.1111/j.1365-3024.2011.01333.x

[16] Naylor C, Burgess S, Madan R, Buonomo E, Razzaq K, Ralston K, et al Leptin Receptor Mutation Results in Defective Neutrophil Recruitment to the Colon during Entamoeba histolytica Infection. mBio. 2014;5(6). . 25516614 10.1128/mBio.02046-14 PMC4271549

[17] Denis M, Chadee K. Human neutrophils activated by interferon-gamma and tumour necrosis factor-alpha kill Entamoeba histolytica trophozoites in vitro. J Leukoc Biol. 1989;46(3):270–4. . 2547889 10.1002/jlb.46.3.270

[18] Zhang D, Chen G, Manwani D, Mortha A, Xu C, Faith JJ, et al Neutrophil ageing is regulated by the microbiome. Nature. 2015;525(7570):528–32. doi: 10.1038/nature15367 . 26374999 10.1038/nature15367 PMC4712631

[19] Kirkpatrick BD, Colgate ER, Mychaleckyj JC, Haque R, Dickson DM, Carmolli MP, et al The "Performance of Rotavirus and Oral Polio Vaccines in Developing Countries" (PROVIDE) study: description of methods of an interventional study designed to explore complex biologic problems. The American journal of tropical medicine and hygiene. 2015;92(4):744–51. doi: 10.4269/ajtmh.14-0518 . 25711607 10.4269/ajtmh.14-0518 PMC4385767

[20] Mondal D, Minak J, Alam M, Liu Y, Dai J, Korpe P, et al Contribution of enteric infection, altered intestinal barrier function, and maternal malnutrition to infant malnutrition in Bangladesh. Clinical infectious diseases: an official publication of the Infectious Diseases Society of America. 2012;54(2):185–92. doi: 10.1093/cid/cir807 . 22109945 10.1093/cid/cir807 PMC3245731

[21] Chassaing B, Srinivasan G, Delgado MA, Young AN, Gewirtz AT, Vijay-Kumar M. Fecal lipocalin 2, a sensitive and broadly dynamic non-invasive biomarker for intestinal inflammation. PloS one. 2012;7(9):e44328 doi: 10.1371/journal.pone.0044328 . 22957064 10.1371/journal.pone.0044328 PMC3434182

[22] Moonah SN, Abhyankar MM, Haque R, Petri WA Jr. The macrophage migration inhibitory factor homolog of Entamoeba histolytica binds to and immunomodulates host macrophages. Infection and immunity. 2014;82(9):3523–30. doi: 10.1128/IAI.01812-14 . 24818664 10.1128/IAI.01812-14 PMC4187827

[23] Kissoon-Singh V, Moreau F, Trusevych E, Chadee K. Entamoeba histolytica exacerbates epithelial tight junction permeability and proinflammatory responses in Muc2(-/-) mice. The American journal of pathology. 2013;182(3):852–65. doi: 10.1016/j.ajpath.2012.11.035 . 23357502 10.1016/j.ajpath.2012.11.035

[24] Noor Z, Watanabe K, Abhyankar MM, Burgess SL, Buonomo EL, Cowardin CA, et al Role of Eosinophils and Tumor Necrosis Factor Alpha in Interleukin-25-Mediated Protection from Amebic Colitis. mBio. 2017;8(1). doi: 10.1128/mBio.02329-16 . 28246365 10.1128/mBio.02329-16 PMC5347349

[25] Zaph C, Du Y, Saenz SA, Nair MG, Perrigoue JG, Taylor BC, et al Commensal-dependent expression of IL-25 regulates the IL-23-IL-17 axis in the intestine. The Journal of experimental medicine. 2008;205(10):2191–8. doi: 10.1084/jem.20080720 . 18762568 10.1084/jem.20080720 PMC2556798

[26] Buonomo EL, Cowardin CA, Wilson MG, Saleh MM, Pramoonjago P, Petri WA Jr. Microbiota-Regulated IL-25 Increases Eosinophil Number to Provide Protection during Clostridium difficile Infection. Cell Rep. 2016;16(2):432–43. doi: 10.1016/j.celrep.2016.06.007 . 27346351 10.1016/j.celrep.2016.06.007 PMC4945404

[27] Mortimer L, Moreau F, Cornick S, Chadee K. The NLRP3 Inflammasome Is a Pathogen Sensor for Invasive Entamoeba histolytica via Activation of alpha5beta1 Integrin at the Macrophage-Amebae Intercellular Junction. PLoS pathogens. 2015;11(5):e1004887 doi: 10.1371/journal.ppat.1004887 . 25955828 10.1371/journal.ppat.1004887 PMC4425650

[28] Mortimer L, Moreau F, Cornick S, Chadee K. Gal-lectin-dependent contact activates the inflammasome by invasive Entamoeba histolytica. Mucosal immunology. 2014;7(4):829–41. doi: 10.1038/mi.2013.100 . 24253103 10.1038/mi.2013.100

[29] Ichinohe T, Pang IK, Kumamoto Y, Peaper DR, Ho JH, Murray TS, et al Microbiota regulates immune defense against respiratory tract influenza A virus infection. Proceedings of the National Academy of Sciences of the United States of America. 2011;108(13):5354–9. doi: 10.1073/pnas.1019378108 . 21402903 10.1073/pnas.1019378108 PMC3069176

[30] Campbell EL, Bruyninckx WJ, Kelly CJ, Glover LE, McNamee EN, Bowers BE, et al Transmigrating neutrophils shape the mucosal microenvironment through localized oxygen depletion to influence resolution of inflammation. Immunity. 2014;40(1):66–77. doi: 10.1016/j.immuni.2013.11.020 . 24412613 10.1016/j.immuni.2013.11.020 PMC3951457

[31] Amulic B, Cazalet C, Hayes GL, Metzler KD, Zychlinsky A. Neutrophil function: from mechanisms to disease. Annu Rev Immunol. 2012;30:459–89. doi: 10.1146/annurev-immunol-020711-074942 . 22224774 10.1146/annurev-immunol-020711-074942

[32] Mehrad B, Strieter RM, Moore TA, Tsai WC, Lira SA, Standiford TJ. CXC chemokine receptor-2 ligands are necessary components of neutrophil-mediated host defense in invasive pulmonary aspergillosis. Journal of immunology. 1999;163(11):6086–94. . 10570298

[33] Arrieta MC, Stiemsma LT, Amenyogbe N, Brown EM, Finlay B. The intestinal microbiome in early life: health and disease. Front Immunol. 2014;5:427 doi: 10.3389/fimmu.2014.00427 . 25250028 10.3389/fimmu.2014.00427 PMC4155789

[34] Kabat AM, Srinivasan N, Maloy KJ. Modulation of immune development and function by intestinal microbiota. Trends Immunol. 2014;35(11):507–17. doi: 10.1016/j.it.2014.07.010 . 25172617 10.1016/j.it.2014.07.010 PMC6485503

[35] Kamada N, Seo SU, Chen GY, Nunez G. Role of the gut microbiota in immunity and inflammatory disease. Nat Rev Immunol. 2013;13(5):321–35. doi: 10.1038/nri3430 . 23618829 10.1038/nri3430

[36] Rakoff-Nahoum S, Paglino J, Eslami-Varzaneh F, Edberg S, Medzhitov R. Recognition of commensal microflora by toll-like receptors is required for intestinal homeostasis. Cell. 2004;118(2):229–41. doi: 10.1016/j.cell.2004.07.002 . 15260992 10.1016/j.cell.2004.07.002

[37] Ivanov II, Frutos Rde L, Manel N, Yoshinaga K, Rifkin DB, Sartor RB, et al Specific microbiota direct the differentiation of IL-17-producing T-helper cells in the mucosa of the small intestine. Cell Host Microbe. 2008;4(4):337–49. doi: 10.1016/j.chom.2008.09.009 . 18854238 10.1016/j.chom.2008.09.009 PMC2597589

[38] Fagarasan S, Muramatsu M, Suzuki K, Nagaoka H, Hiai H, Honjo T. Critical roles of activation-induced cytidine deaminase in the homeostasis of gut flora. Science. 2002;298(5597):1424–7. doi: 10.1126/science.1077336 . 12434060 10.1126/science.1077336

[39] Elinav E, Strowig T, Kau AL, Henao-Mejia J, Thaiss CA, Booth CJ, et al NLRP6 inflammasome regulates colonic microbial ecology and risk for colitis. Cell. 2011;145(5):745–57. doi: 10.1016/j.cell.2011.04.022 . 21565393 10.1016/j.cell.2011.04.022 PMC3140910

[40] Seo SU, Kamada N, Munoz-Planillo R, Kim YG, Kim D, Koizumi Y, et al Distinct Commensals Induce Interleukin-1beta via NLRP3 Inflammasome in Inflammatory Monocytes to Promote Intestinal Inflammation in Response to Injury. Immunity. 2015;42(4):744–55. doi: 10.1016/j.immuni.2015.03.004 . 25862092 10.1016/j.immuni.2015.03.004 PMC4408263

[41] Houpt ER, Glembocki DJ, Obrig TG, Moskaluk CA, Lockhart LA, Wright RL, et al The mouse model of amebic colitis reveals mouse strain susceptibility to infection and exacerbation of disease by CD4+ T cells. Journal of immunology. 2002;169(8):4496–503. . 12370386 10.4049/jimmunol.169.8.4496

[42] Noor Z, Watanabe K, Abhyankar MM, Burgess SL, Buonomo EL, Cowardin CA, et al IL-25—Mediated Protection from Amebic Colitis: Role of Eosinophils and TNF-α. mBio. 2017;in press. 10.1128/mBio.02329-16 PMC5347349 28246365

[43] De Filippo K, Henderson RB, Laschinger M, Hogg N. Neutrophil chemokines KC and macrophage-inflammatory protein-2 are newly synthesized by tissue macrophages using distinct TLR signaling pathways. Journal of immunology. 2008;180(6):4308–15. . 18322244 10.4049/jimmunol.180.6.4308

[44] Soehnlein O, Lindbom L. Phagocyte partnership during the onset and resolution of inflammation. Nat Rev Immunol. 2010;10(6):427–39. doi: 10.1038/nri2779 . 20498669 10.1038/nri2779

[45] de Oliveira S, Rosowski EE, Huttenlocher A. Neutrophil migration in infection and wound repair: going forward in reverse. Nat Rev Immunol. 2016;16(6):378–91. doi: 10.1038/nri.2016.49 . 27231052 10.1038/nri.2016.49 PMC5367630

[46] Moore TA, Newstead MW, Strieter RM, Mehrad B, Beaman BL, Standiford TJ. Bacterial clearance and survival are dependent on CXC chemokine receptor-2 ligands in a murine model of pulmonary Nocardia asteroides infection. Journal of immunology. 2000;164(2):908–15. . 10623839 10.4049/jimmunol.164.2.908

[47] Tsai WC, Strieter RM, Mehrad B, Newstead MW, Zeng X, Standiford TJ. CXC chemokine receptor CXCR2 is essential for protective innate host response in murine Pseudomonas aeruginosa pneumonia. Infection and immunity. 2000;68(7):4289–96. . 10858247 10.1128/iai.68.7.4289-4296.2000 PMC101748

[48] Spehlmann ME, Dann SM, Hruz P, Hanson E, McCole DF, Eckmann L. CXCR2-dependent mucosal neutrophil influx protects against colitis-associated diarrhea caused by an attaching/effacing lesion-forming bacterial pathogen. Journal of immunology. 2009;183(5):3332–43. doi: 10.4049/jimmunol.0900600 . 19675161 10.4049/jimmunol.0900600 PMC3419829

[49] Boppana NB, Devarajan A, Gopal K, Barathan M, Bakar SA, Shankar EM, et al Blockade of CXCR2 signalling: a potential therapeutic target for preventing neutrophil-mediated inflammatory diseases. Exp Biol Med (Maywood). 2014;239(5):509–18. doi: 10.1177/1535370213520110 . 24625439 10.1177/1535370213520110

[50] Nguyen TL, Vieira-Silva S, Liston A, Raes J. How informative is the mouse for human gut microbiota research? Dis Model Mech. 2015;8(1):1–16. doi: 10.1242/dmm.017400 . 25561744 10.1242/dmm.017400 PMC4283646

[51] Ivanov II, Atarashi K, Manel N, Brodie EL, Shima T, Karaoz U, et al Induction of intestinal Th17 cells by segmented filamentous bacteria. Cell. 2009;139(3):485–98. doi: 10.1016/j.cell.2009.09.033 . 19836068 10.1016/j.cell.2009.09.033 PMC2796826

[52] Nomellini V, Faunce DE, Gomez CR, Kovacs EJ. An age-associated increase in pulmonary inflammation after burn injury is abrogated by CXCR2 inhibition. J Leukoc Biol. 2008;83(6):1493–501. doi: 10.1189/jlb.1007672 . 18319289 10.1189/jlb.1007672 PMC6615035

[53] Hamano S, Asgharpour A, Stroup SE, Wynn TA, Leiter EH, Houpt E. Resistance of C57BL/6 mice to amoebiasis is mediated by nonhemopoietic cells but requires hemopoietic IL-10 production. Journal of immunology. 2006;177(2):1208–13. . 16818779 10.4049/jimmunol.177.2.1208

[54] Cowardin CA, Buonomo EL, Saleh MM, Wilson MG, Burgess SL, Kuehne SA, et al The binary toxin CDT enhances Clostridium difficile virulence by suppressing protective colonic eosinophilia. Nat Microbiol. 2016;1(8):16108 doi: 10.1038/nmicrobiol.2016.108 . 27573114 10.1038/nmicrobiol.2016.108 PMC5010011

[55] Chassaing B, Koren O, Goodrich JK, Poole AC, Srinivasan S, Ley RE, et al Dietary emulsifiers impact the mouse gut microbiota promoting colitis and metabolic syndrome. Nature. 2015;519(7541):92–6. doi: 10.1038/nature14232 . 25731162 10.1038/nature14232 PMC4910713

[56] Wirtz S, Neufert C, Weigmann B, Neurath MF. Chemically induced mouse models of intestinal inflammation. Nat Protoc. 2007;2(3):541–6. doi: 10.1038/nprot.2007.41 . 17406617 10.1038/nprot.2007.41

[57] Dharmani P, Leung P, Chadee K. Tumor necrosis factor-alpha and Muc2 mucin play major roles in disease onset and progression in dextran sodium sulphate-induced colitis. PloS one. 2011;6(9):e25058 doi: 10.1371/journal.pone.0025058 . 21949848 10.1371/journal.pone.0025058 PMC3176316

[58] Verweij JJ, Oostvogel F, Brienen EA, Nang-Beifubah A, Ziem J, Polderman AM. Short communication: Prevalence of Entamoeba histolytica and Entamoeba dispar in northern Ghana. Trop Med Int Health. 2003;8(12):1153–6. . 14641852 10.1046/j.1360-2276.2003.01145.x

[59] Liu J, Kabir F, Manneh J, Lertsethtakarn P, Begum S, Gratz J, et al Development and assessment of molecular diagnostic tests for 15 enteropathogens causing childhood diarrhoea: a multicentre study. Lancet Infect Dis. 2014;14(8):716–24. doi: 10.1016/S1473-3099(14)70808-4 . 25022434 10.1016/S1473-3099(14)70808-4

[60] Faith JJ, Guruge JL, Charbonneau M, Subramanian S, Seedorf H, Goodman AL, et al The long-term stability of the human gut microbiota. Science. 2013;341(6141):1237439 doi: 10.1126/science.1237439 . 23828941 10.1126/science.1237439 PMC3791589

[61] Caporaso JG, Lauber CL, Walters WA, Berg-Lyons D, Huntley J, Fierer N, et al Ultra-high-throughput microbial community analysis on the Illumina HiSeq and MiSeq platforms. ISME J. 2012;6(8):1621–4. doi: 10.1038/ismej.2012.8 . 22402401 10.1038/ismej.2012.8 PMC3400413

[62] Beck D, Dennis C, Foster JA. Seed: a user-friendly tool for exploring and visualizing microbial community data. Bioinformatics. 2015;31(4):602–3. doi: 10.1093/bioinformatics/btu693 . 25332377 10.1093/bioinformatics/btu693 PMC4325548

[63] Geem D, Medina-Contreras O, Kim W, Huang CS, Denning TL. Isolation and characterization of dendritic cells and macrophages from the mouse intestine. J Vis Exp. 2012;(63):e4040 doi: 10.3791/4040 . 22644046 10.3791/4040 PMC3466926

