# Supplementary material for: Microbiome-mediated neutrophil recruitment via CXCR2 and protection from amebic colitis
Source: PLoS Pathog. 2017 Aug 17;13(8):e1006513. doi: 10.1371/journal.ppat.1006513 (PMC5560520; doi:10.1371/journal.ppat.1006513)
Supplement: S1 Fig — The Shannon diversity index was examined using stool samples collected from children followed longitudinally in an urban slum in Dhaka, Bangladesh who developed amebic colitis within the first 2 years of life (n = 18). The Shannon diversity index was measured one month prior to amebic colitis, and compared to that from children who did not develop E. histolytica infection (n = 72). *P<0.05, by Welch’s unequal variance t-test. Error bars represent standard error of the mean (s.e.m). (PDF) [file ppat.1006513.s001.pdf]

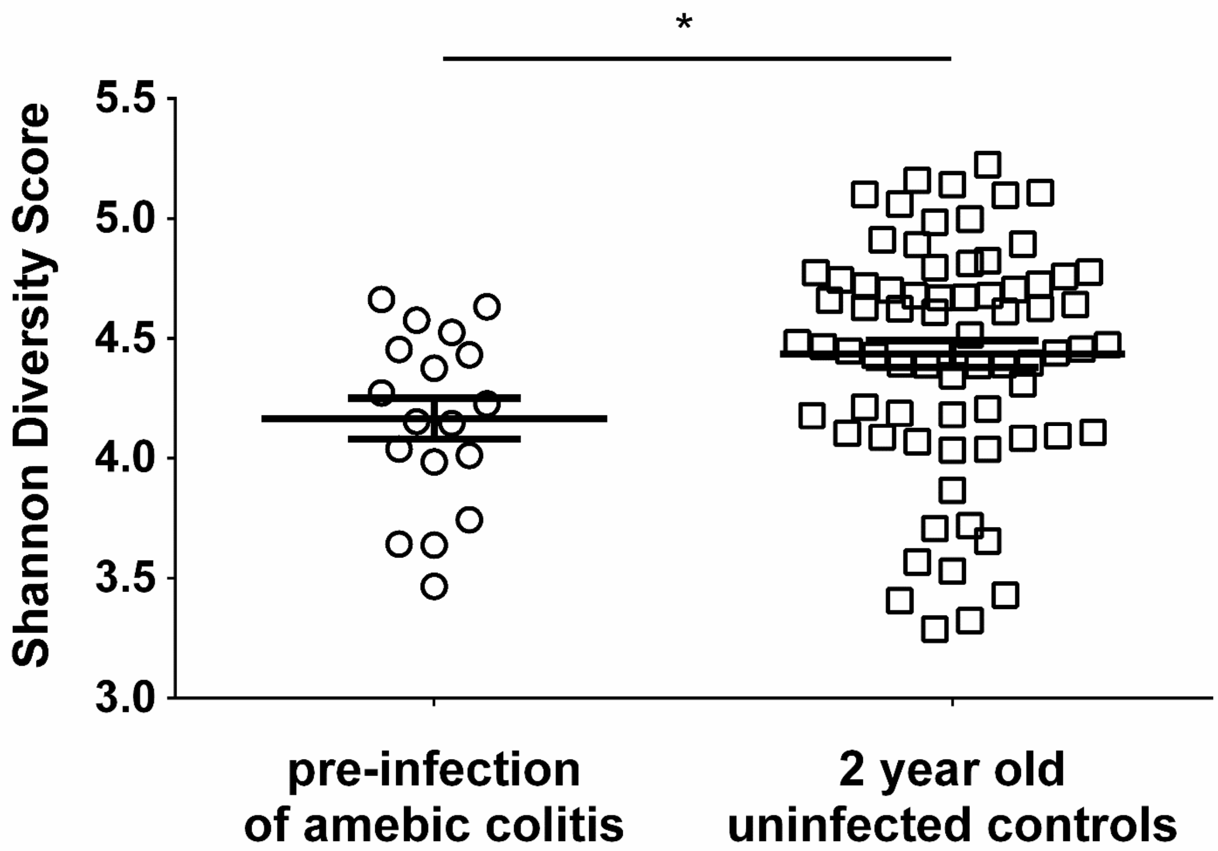

S1 Fig. Diversity of microbiome was decreased prior to amebic colitis. The Shannon diversity index was examined using stool samples collected from children followed longitudinally in an urban slum in Dhaka, Bangladesh who developed amebic colitis within the first 2 years of life (n=18). The Shannon diversity index was measured one month prior to amebic colitis, and compared to that from children who did not develop *E. histolytica* infection (n=72). **P<0.05,* by Welch's unequal variance t-test. Error bars represent standard error of the mean (s.e.m).
