# Supplementary material for: Microbiome-mediated neutrophil recruitment via CXCR2 and protection from amebic colitis
Source: PLoS Pathog. 2017 Aug 17;13(8):e1006513. doi: 10.1371/journal.ppat.1006513 (PMC5560520; doi:10.1371/journal.ppat.1006513)
Supplement: S2 Fig — Histopathological examinations by hematoxilin and eosin stain were performed using cecal tissue collected from mice sacrificed at 24 hours after E. histolytica challenge. Yellow arrows represent E. histolytica. (a, b) Representative pictures of tissue invasion by E. histolytica in antibiotic pretreated mice. (c, d) Representative pictures of ulcerative lesions in untreated control mice. (a, c) x200 represents yellow boxes in a and d, (b, d) x400 represents yellow boxes in a and c. (PDF) [file ppat.1006513.s002.pdf]

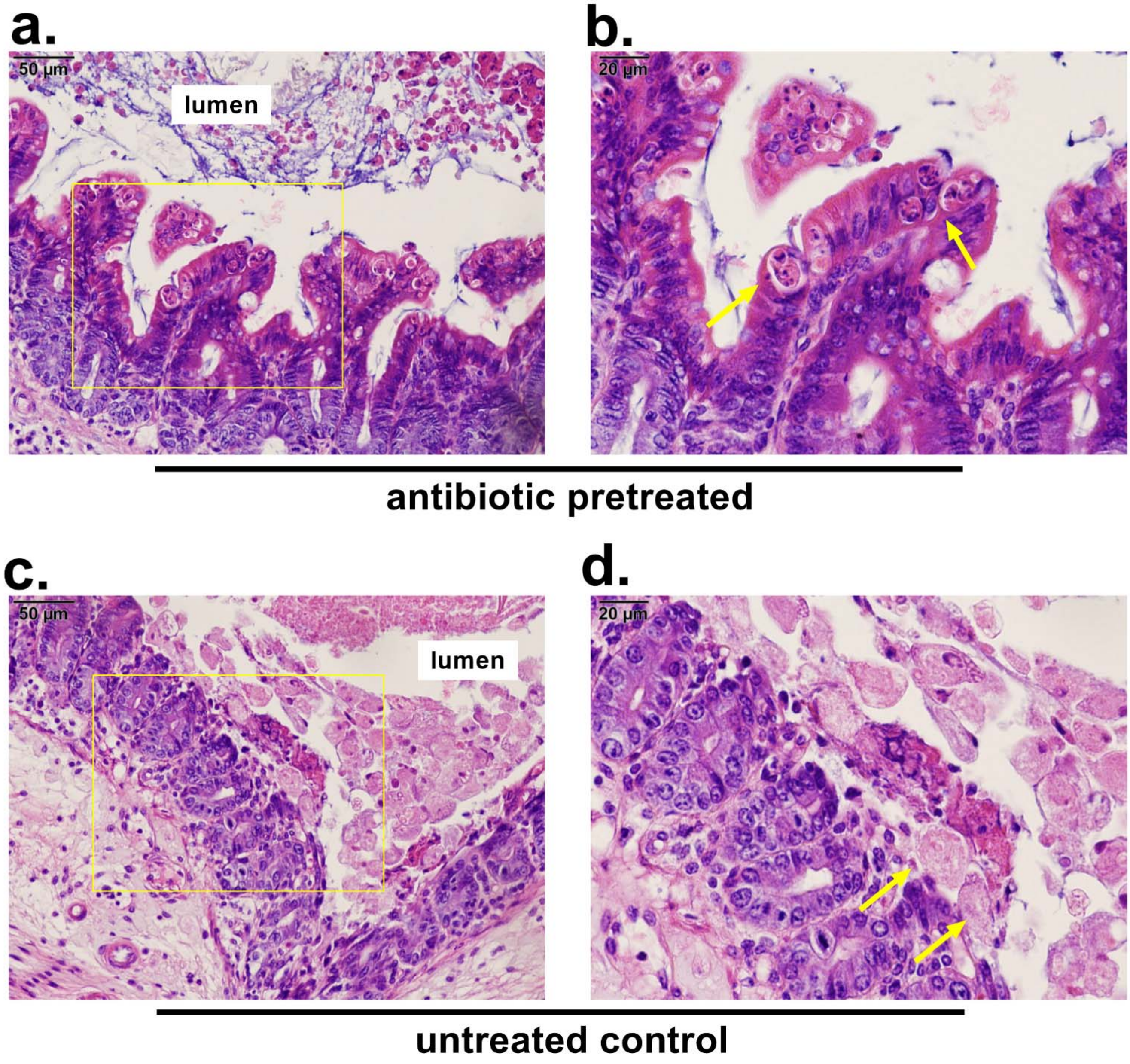


**S2 Fig. Tissue invasion into intestinal epithelial cells by *E. histolytica* in antibiotic pre-treated mice but not in untreated control mice**. Histopathological examinations by hematoxilin and eosin stain were performed using cecal tissue collected from mice sacrificed at 24 hours after *E. histolytica* challenge. Yellow arrows represent *E. histolytica*. (**a, b)** Representative pictures of tissue invasion by *E. histolytica* in antibiotic pretreated mice. **(c, d)** Representative pictures of ulcerative lesions in untreated control mice. **(a, c)** x200 represents yellow boxes in a and d, **(b, d)** x400 represents yellow boxes in  a and c.
