# Supplementary material for: Microbiome-mediated neutrophil recruitment via CXCR2 and protection from amebic colitis
Source: PLoS Pathog. 2017 Aug 17;13(8):e1006513. doi: 10.1371/journal.ppat.1006513 (PMC5560520; doi:10.1371/journal.ppat.1006513)
Supplement: S3 Fig — Antibiotic pre-treated or untreated control wild type C57BL/6 mice were infected with 2 x 10^6^ E. histolytica trophozoites intracecally, and were sacrificed at day 9. (a) E. histoltyica burden was measured by qPCR. (b, c) lipocalin-2 and anti-lectin IgA were assessed by ELISA using 200 μL of cecal contents. Data are representative from similarly conducted two independent experiments. *P<0.05, **P<0.01, ***P<0.001 by Welch’s unequal variance. NS, not significant. Error bars represent s.e.m. (PDF) [file ppat.1006513.s003.pdf]

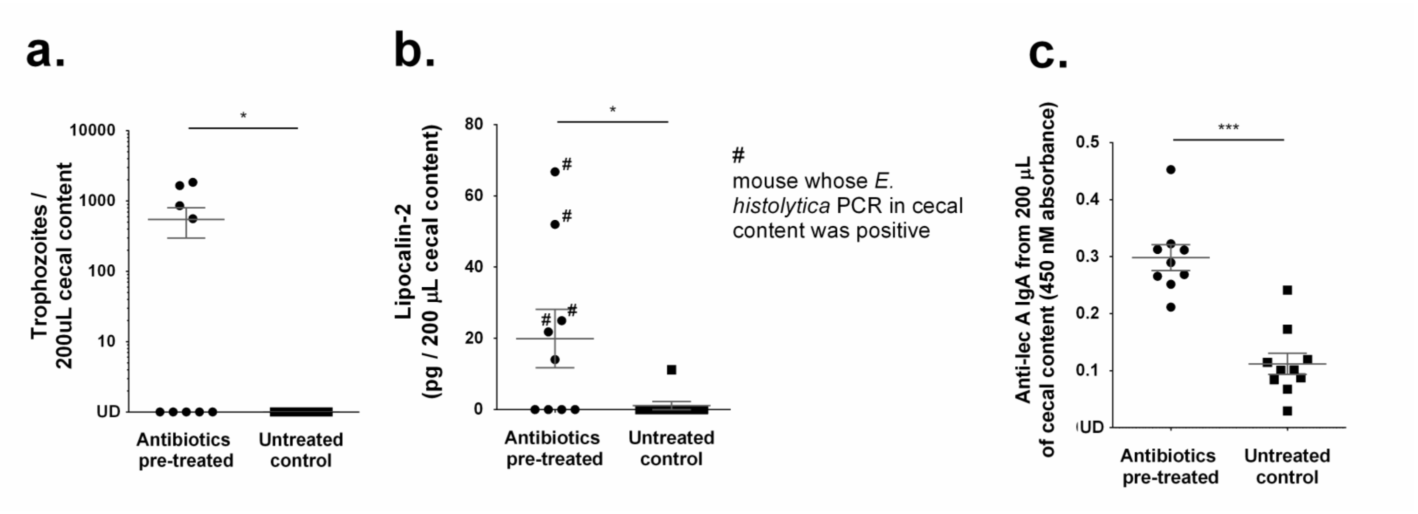

S3 Fig. Immune responses at day 9 are correlated with Infection outcome of *E. histolytica*. Antibiotic pre-treated or untreated control wild type C57BL/6 mice were infected with 2 x 10^6 *E. histolytica* trophozoites intracecally, and were sacrificed at day 9. **(a)** *E. histoltyica* burden was measured by qPCR. **(b, c)** lipocalin-2 and anti-lectin IgA were assessed by ELISA using 200 μL of cecal contents. Data are representative from similarly conducted two independent experiments. **P<0.05, **P<0.01, ***P<0.001* by Welch's unequal variance. NS, not significant. Error bars represent s.e.m.
