# Supplementary material for: Microbiome-mediated neutrophil recruitment via CXCR2 and protection from amebic colitis
Source: PLoS Pathog. 2017 Aug 17;13(8):e1006513. doi: 10.1371/journal.ppat.1006513 (PMC5560520; doi:10.1371/journal.ppat.1006513)
Supplement: S4 Fig — Antibiotic pre-treated or untreated control wild type C57BL/6 mice were sacrificed at 2 weeks of antibiotics in order to see the baseline data of IL-1β, CXCL1 and CXCL2 in cecal tissue before E. histolytica challenge. Cecal cytokines were assessed by lysing 50mg of cecal sections and quantifying protein via ELISA, and shown normalized to total protein concentration (data from single experiment, n = 5 per group). *P<0.05, **P<0.01, ***P<0.001 by Welch’s unequal variance. NS, not significant. Error bars represent s.e.m. (PDF) [file ppat.1006513.s004.pdf]

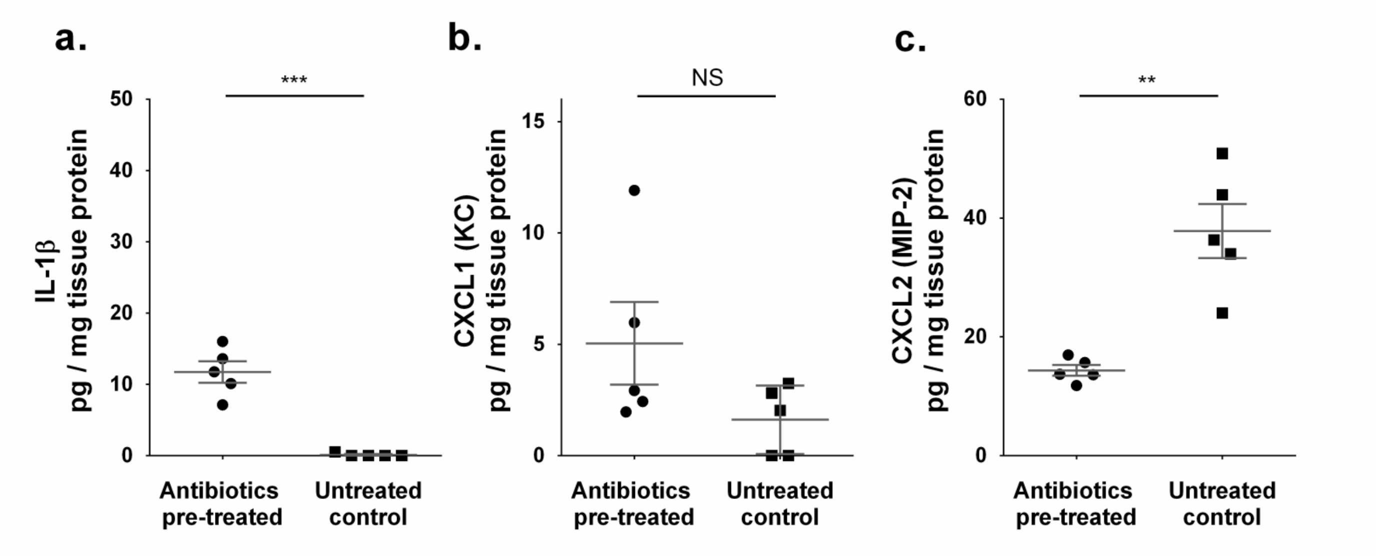

S4 Fig. IL-1β and neutrophil attractant chemokines at baseline. Antibiotic pre-treated or untreated control wild type C57BL/6 mice were sacrificed at 2 weeks of antibiotics in order to see the baseline data of IL-1β, CXCL1 and CXCL2 in cecal tissue before *E. histolytica* challenge. Cecal cytokines were assessed by lysing 50mg of cecal sections and quantifying protein via ELISA, and shown normalized to total protein concentration (data from single experiment, n=5 per group). **P<0.05, **P<0.01, ***P<0.001* by Welch's unequal variance. NS, not significant. Error bars represent s.e.m.
