# Supplementary material for: Microbiome-mediated neutrophil recruitment via CXCR2 and protection from amebic colitis
Source: PLoS Pathog. 2017 Aug 17;13(8):e1006513. doi: 10.1371/journal.ppat.1006513 (PMC5560520; doi:10.1371/journal.ppat.1006513)
Supplement: S5 Fig — Antibiotic pre-treated or untreated control wild type C57BL/6 mice were infected with 2 x 10^6^ E. histolytica trophozoites intracecally. Surface protein expression levels were assessed as mean fluorescence intensity (MFI) by flow cytometry using single cell suspension from blood and lamina propria. (a, b) Surface protein expression levels before E. histolytica challenge. (c, d) Surface protein expression levels at 24 hours after E. histolytica challenge. Data are from a single experiment, n = 8 per group. *P<0.05, **P<0.01, ***P<0.001 by Welch’s unequal variance t-test NS, not significant. Error bars represent s.e.m. (PDF) [file ppat.1006513.s005.pdf]

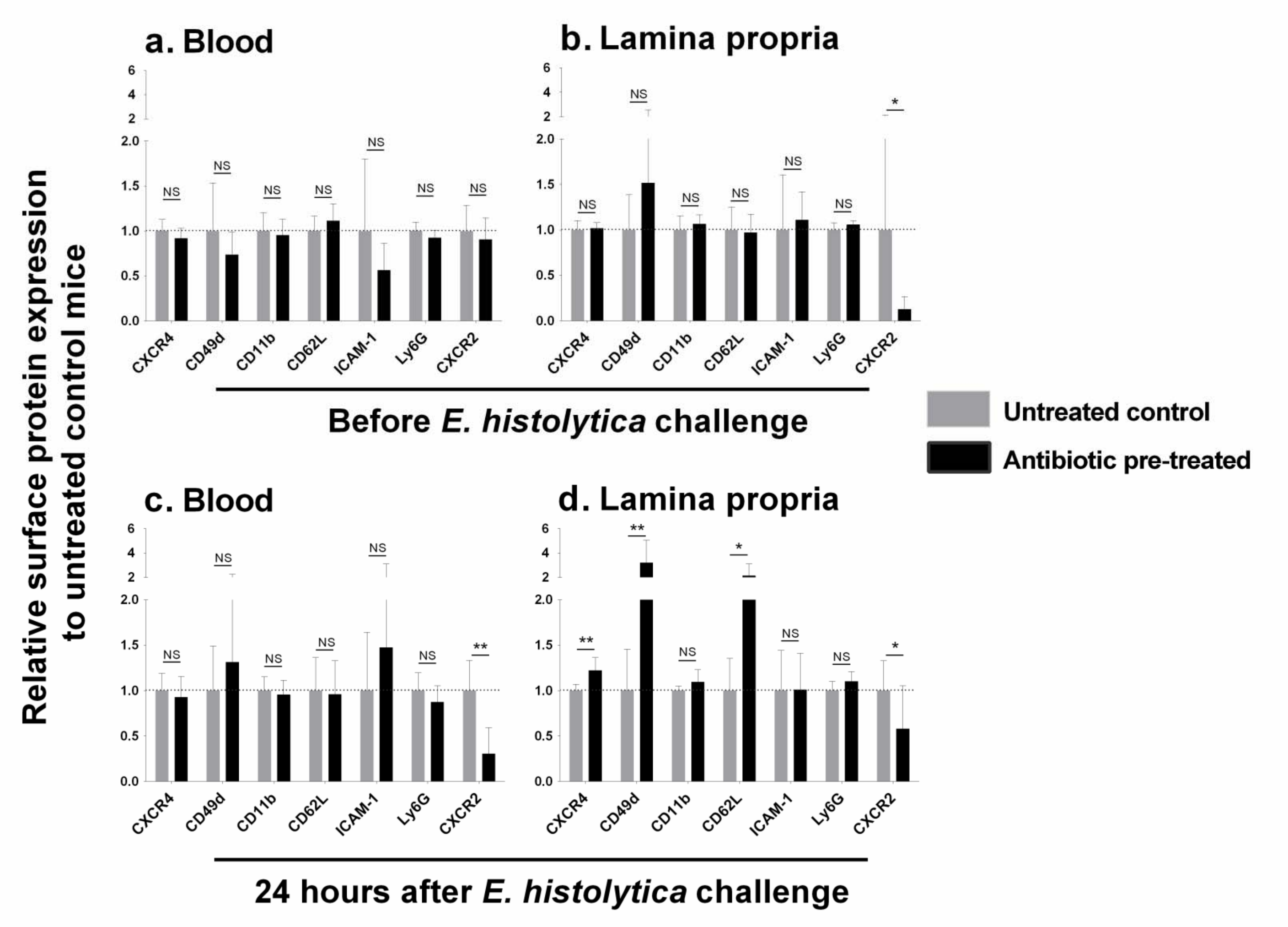

S5 Fig. Surface expression of molecules on neutrophils. Antibiotic pre-treated or untreated control wild type C57BL/6 mice were infected with 2 x 10^6 *E. histolytica* trophozoites intracecally. Surface protein expression levels were assessed as mean fluorescence intensity (MFI) by flow cytometry using single cell suspension from blood and lamina propria. **(a, b)** Surface protein expression levels before *E. histolytica* challenge. **(c, d)** Surface protein expression levels at 24 hours after *E. histolytica challenge.* Data are from a single experiment, n=8 per group. **P<0.05, **P<0.01, ***P<0.001* by Welch's unequal variance t-test NS, not significant. Error bars represent s.e.m.
