# Supplementary material for: Microbiome-mediated neutrophil recruitment via CXCR2 and protection from amebic colitis
Source: PLoS Pathog. 2017 Aug 17;13(8):e1006513. doi: 10.1371/journal.ppat.1006513 (PMC5560520; doi:10.1371/journal.ppat.1006513)
Supplement: S1 Table — (PDF) [file ppat.1006513.s006.pdf]

S1 Table. Clinical Scoring

	0	1	2	3	4
Weight loss	No weight loss	< 10% Weight loss	10–15% weight loss	15–20% Weight loss	> 20% Weight loss
Coat	Normal	Slightly ruffled	Rough/Hair loss	Very ruffled/puff	
Eyes/Nose	Normal	Squinted 1/2 closed	Squinted/discharge	Closed/discharge	
Activity	Normal	Alert/slow moving	Lethargic/shaky	Inactive/Unless prodded	Not moving
Posture	Normal	Back slanted	Hunched	Hunched Nosedown	
Diarrhea	Normal	Soft stool Discolored (Yellow)	Wet Stained tail/mucous	Liquid or no stool	

Clinical srore: sum of all parameter scores, Normal = 0, Total possible score = 20, Found dead = 20
