# Supplementary material for: Microbiome-mediated neutrophil recruitment via CXCR2 and protection from amebic colitis
Source: PLoS Pathog. 2017 Aug 17;13(8):e1006513. doi: 10.1371/journal.ppat.1006513 (PMC5560520; doi:10.1371/journal.ppat.1006513)
Supplement: S2 Table — (PDF) [file ppat.1006513.s007.pdf]

S2 Table. Conjugated Antibodies used for Flowcytometry

Fluorochrome	Antibody	Source	Clone
Brilliant Violet 421	CD11c	Biolegend	N418
Brilliant Violet 605	CD62L	Biolegend	MEL-14
FITC	CXCR2	Biolegend	SA044G4
PerCP-Cy5.5	CXCR4	Biolegend	L276F12
PE	SiglecF	BD Pharmingen	E50-2440
PE	CD54 (ICAM-1)	Biolegend	YN1/1.7.4
PE-Cy7	Ly6G	Biolegend	1A8
APC	CD11b	Biolegend	M1/70
APC	CD49d	Biolegend	R1-2
AlexaFluor 700	Ly6c	Biolegend	HK1.4
AlexaFluor 700	CD11b	Biolegend	M1/70
APC-Cy7	CD45	Biolegend	30-F11
